# Origin and evolution of spliceosomal introns

**DOI:** 10.1186/1745-6150-7-11

**Published:** 2012-04-16

**Authors:** Igor B Rogozin, Liran Carmel, Miklos Csuros, Eugene V Koonin

**Affiliations:** 1 National Center for Biotechnology Information NLM/NIH, 8600 Rockville Pike, Bldg. 38A, Bethesda, MD, 20894, USA; 2 Department of Genetics, The Alexander Silberman Institute of Life Sciences, The Hebrew University of Jerusalem, Edmund J. Safra Campus, Givat Ram, Jerusalem, 91904, Israel; 3 Department of Computer Science and Operations Research, Université de Montréal, Montréal, Québec, Canada

**Keywords:** Intron sliding, Intron gain, Intron loss, Spliceosome, Splicing signals, Evolution of exon/intron structure, Alternative splicing, Phylogenetic trees, Mobile domains, Eukaryotic ancestor

## Abstract

Evolution of exon-intron structure of eukaryotic genes has been a matter of long-standing, intensive debate. The introns-early concept, later rebranded ‘introns first’ held that protein-coding genes were interrupted by numerous introns even at the earliest stages of life's evolution and that introns played a major role in the origin of proteins by facilitating recombination of sequences coding for small protein/peptide modules. The introns-late concept held that introns emerged only in eukaryotes and new introns have been accumulating continuously throughout eukaryotic evolution. Analysis of orthologous genes from completely sequenced eukaryotic genomes revealed numerous shared intron positions in orthologous genes from animals and plants and even between animals, plants and protists, suggesting that many ancestral introns have persisted since the last eukaryotic common ancestor (LECA). Reconstructions of intron gain and loss using the growing collection of genomes of diverse eukaryotes and increasingly advanced probabilistic models convincingly show that the LECA and the ancestors of each eukaryotic supergroup had intron-rich genes, with intron densities comparable to those in the most intron-rich modern genomes such as those of vertebrates. The subsequent evolution in most lineages of eukaryotes involved primarily loss of introns, with only a few episodes of substantial intron gain that might have accompanied major evolutionary innovations such as the origin of metazoa. The original invasion of self-splicing Group II introns, presumably originating from the mitochondrial endosymbiont, into the genome of the emerging eukaryote might have been a key factor of eukaryogenesis that in particular triggered the origin of endomembranes and the nucleus. Conversely, splicing errors gave rise to alternative splicing, a major contribution to the biological complexity of multicellular eukaryotes. There is no indication that any prokaryote has ever possessed a spliceosome or introns in protein-coding genes, other than relatively rare mobile self-splicing introns. Thus, the introns-first scenario is not supported by any evidence but exon-intron structure of protein-coding genes appears to have evolved concomitantly with the eukaryotic cell, and introns were a major factor of evolution throughout the history of eukaryotes. This article was reviewed by I. King Jordan, Manuel Irimia (nominated by Anthony Poole), Tobias Mourier (nominated by Anthony Poole), and Fyodor Kondrashov. For the complete reports, see the Reviewers’ Reports section.

## Genes in pieces: exon-intron structure of eukaryotic genes and the two spliceosomes

In a memorable phrase of Walter Gilbert, eukaryotes possess “genes in pieces” in which protein-coding sequences are interrupted by non-coding sequences denoted introns [1]. The introns are excised at the donor and acceptor splice sites such that the flanking coding regions, exons, are spliced by an extremely complex ribonucleoprotein molecular machine, the spliceosome [2,3]. Multiple introns interrupt the coding sequences in the great majority of genes in animals and plants, whereas intron densities in fungi and unicellular eukaryotes are highly variable: many of the unicellular forms contain only a few introns in the entire genome whereas in others the intron density approaches that in animals and plants [4-6]. Remarkably, however, there is no sequenced genome of a full-fledged eukaryote without introns at all; only one intronless genome of a highly degraded remnant of a eukaryotic organism, a nucleomorph that has also lost the genes for the spliceosome subunits, has been reported [7].

**Figure 1:**
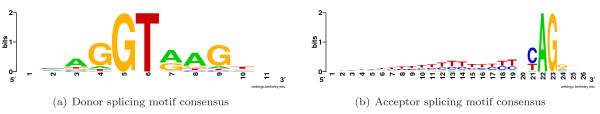
**Consensus motifs for donor and acceptor splicing signals.** The Y axis indicates the strength of splicing signals (base composition bias based on information content). The data is from [19].

The ubiquity of introns in eukaryotes is complemented by the conservation of the spliceosome. The spliceosome consists of five snRNPs (small nuclear ribonucleoprotein particles), together with numerous less stably associated proteins; the core of the spliceosome is conserved in all well-characterized eukaryotes [2,3,8]. The spliceosome interacts with specific sites in the intron and the flanking exons to ensure accurate and efficient splicing. The nucleotides at the intron termini and the adjacent nucleotides in the exons are involved in these interactions and comprise the splicing signals. The (A/C)AG|GU(A/G)AGU sequence (the splice site is shown by the vertical streak and the first two nucleotides of the intron are underlined) at the donor splice signal is complementary to the 5’ end of the U1 snRNA, and this interaction appears to be the major requirement for splicing [9-11]. The (C,U)AG|G sequence (the last two nucleotides of the intron are underlined) preceded by a polypyrimidine tract is typical of the acceptor splice signal (Figure 1) and is recognized by the U5 snRNA [12,13]. A short branch point signal is located in the intron sequence upstream of the acceptor splice signals and contains the reactive adenosine that is involved in the formation of the lariat-like structure in the splicing intermediate [12,13]. The functionally important (A/C)AG||G exon sequences flanking introns have been dubbed protosplice sites with the implication that new introns insert into sites of this structure [14,15]. Some lineage-specific deviations from the canonical variants of splice signals are known to exist. For example, some unicellular eukaryotes lack recognizable polyT tracts between the branch point signal and the 3’ splice signal [16,17]. Some extremely intron-poor species such as yeast possess an unusual, strictly constrained donor splice signal |GTA(T,A,C)G(T,A,C) with a substantial excess of T at position +4 [16-18]. 

The vast majority of spliceosomal introns contain |GT at the donor splice site and AG| at the acceptor splice site. However, a distinct class of rare introns has been recognized on the basis of their unusual terminal dinucleotides: these introns contain |AT at the donor splice site and AC| at the acceptor splice site [20,21]. A closer examination of the sequences of these atypical introns revealed several properties that distinguish them from the majority of the introns including conservation of unusual signals at the donor splice signal (|ATATCCTT) and immediately upstream of the acceptor splice signal (TCCTTAAC 10-15 bases from the splice junction) [20,21]. Introns of this class are excised by a distinct, so-called minor or U12 spliceosome, which contains several specific, low-abundance snRNPs. It has been subsequently shown that some |GT-AG| introns are also removed by the U12 spliceosome [22]. The U12 introns and the associated minor spliceosome are not universally conserved, like the major U2 spliceosome, but are also widespread in eukaryotes, being represented in vertebrates, insects, plants, and some protists [23-26].

Phylogenomic reconstructions for the small RNA and protein subunits of the U2 and U12 spliceosomes suggest that both spliceosomes were already present in the last common ancestor of the extant eukaryotes (LECA, Last Eukaryotic Common Ancestor) as a result of ancient duplication of the genes for the respective components [24]. Taking into account a potentially important role of U12 introns in regulation of gene expression [27-29], it might be tempting to speculate that the ancestral introns were of the U12 type (for example, see discussion by the reviewer #3 below) but have been subsequently converted to U2 introns. However, comparison of protosplice sites (exonic sequences surrounding introns) of ancient U2 and U12 introns in human and Arabidopsis revealed close similarity of ancestral introns to U2 but not to U12. Thus, the primordial spliceosomal introns were most likely of the U2-type [30].

The two principal mechanisms of splicing signal recognition are known as exon definition and intron definition [31-34]. Evidence of these two mechanisms has come from analyses of interactions between pre-mRNAs and various splicing factors [32,33,35]. The exon definition mechanism involves SR proteins binding to exonic splicing enhancers (ESE) and recruiting U1 to the downstream donor splicing signal and the splicing factor U2AF to the upstream acceptor splicing signal. The U2AF factor then recruits U2 to the branch site. Therefore, when the SR proteins bind the ESEs, they promote formation of a “cross-exon” recognition complex by placing the basal splicing machinery at the splice sites flanking the same exon. The intron definition mechanism requires binding of U1 to the upstream donor splice site and binding of U2AF/U2 to the downstream acceptor splice signal and branch site, respectively, of the same intron. Therefore, intron definition selects pairs of splice sites located on both ends of the same intron, and SR proteins can also mediate this process [32,36]. The efficiency of splicing under the exon definition depends on the length of exons but is not affected by the length of introns; conversely, under the intron definition, the efficiency of splicing depends on the length of introns, but not that of exons [31-35,37].

## Introns-early, introns-late, introns-first: the competing scenarios of intron origin and evolution

Evolution of exon-intron structure of eukaryotic genes and evolutionary properties of introns had been long considered in the context of the “introns-early” vs. “introns-late” debate [38-42]. The original, “strong” introns-early hypothesis held that eukaryotic genes inherited (nearly) all introns from prokaryotic ancestors and that the differences in gene structure among homologous eukaryotic genes were due mostly to differential intron loss [39]. Under this scenario, the extant prokaryotes have lost all the primordial introns and the spliceosome in the process of ‘genome streamlining’. The later adaptations of the introns-early hypothesis assumed an intermediate position by allowing emergence of some new introns, in addition to the ancient ones [40]. The introns-late concept countered that introns were a eukaryotic novelty and new introns have been emerging continuously throughout eukaryotic evolution; in this scenario, bacteria and archaea never possessed intron or the spliceosome [41,43,44]. These hypotheses have been later merged into a synthetic concept that can be denoted ‘many introns early in eukaryotic evolution’ [45,46] and that we discuss in greater detail below. In addition, there has been an attempt to revitalize the introns early idea in the ‘introns first’ scenario according to which exons of protein-coding genes emerged from the primordial introns, i.e. non-coding regions that are presumed to have been interspersed between functional RNA sequences in the genes that existed in the RNA world and antedated proteins [47,48].

## Intron density, size and distribution in protein-coding genes across the eukaryote domain

**Figure 2:**
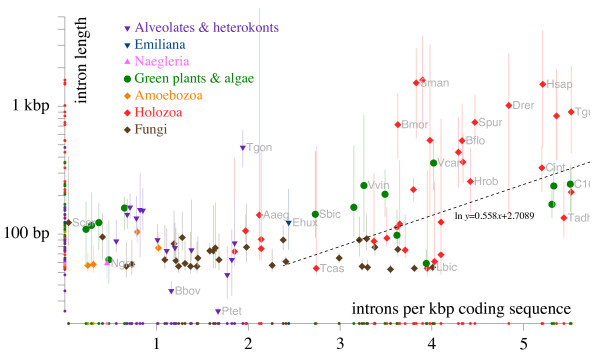
**Intron density and intron length in 100 eukaryotes.** The data is from [53].

Genes of eukaryotes from different groups dramatically differ in intron density and size distribution, from only a few introns in the entire genome (that is, near zero density per gene or per kilobase) in many unicellular organisms to approximately 6 introns per kilobase (kb) of coding sequence in mammals (Figure 2). With respect to intron content, eukaryotic genomes are often crudely classified into intron-poor ones (most unicellular forms) and intron-rich ones including animals, plants, some fungi, and a few unicellular organisms such as *Chlamydomonas* or some diatoms (Figure 2) [42,49-52]. Although this division is appealing in its simplicity and may be convenient for the purpose of various comparative analyses, examination of intron densities in 100 sequenced eukaryotic genomes does not present an obvious bimodal distribution (Figure 2). Actually, it appears that all intron densities between 0 and 6 introns per kilobase are observed in some eukaryote genomes. However, when intron density is plotted against intron length, partitioning of eukaryote genomes into two classes becomes apparent. While up to a density of approximately 3 introns per kilobase, all introns are short, with no significant correlation between the density and length of introns, for more intron-rich genomes, a strong positive correlation is observed (linear correlation coefficient = 0.16, P = 0.003, Figure 2). Even among intron-rich organisms, vertebrates are outstanding in having a substantial fraction of extremely long introns (Figure 2). This strong correlation notwithstanding, there are exceptions to the general trend: intron-rich basidiomycete fungi (3-4 introns/kbp) have only short introns whereas some insects show broad intron length distributions with multiple long introns despite relatively low intron density (2-3 introns/kbp) (Figure 2). We return to the dependencies between intron density, intron length and structure of splice signals later, in the discussion of the selection pressures affecting the evolution of eukaryote gene architecture and the underlying population-genetic factors. 

As pointed out above, despite the existence of numerous, diverse intron-poor genomes, eukaryotes do not lose the “last” intron or the spliceosome although degradation of the spliceosome including loss of many components does occur, e.g. in yeast. The only firmly established exception is the tiny genome of a nucleomorph (an extremely degraded intracellular symbiont of algae) that has lost both all the introns and the spliceosome [7]; preliminary genomic data indicate that all introns might have been lost also in a microsporidium, a highly degraded intracellular parasite distantly related to Fungi [54]. In general, it remains unclear whether there are any selective factors or functional constraint underpinning this surprising preservation of at least a few introns in eukaryote genomes [55]. However, in many cases, the few introns that are retained in highly reduced genomes are present in 5’-portions of genes encoding ribosomal proteins [16,56]. The introns in these genes are important for regulation of expression and ribosomal biogenesis, and their deletion leads to significant fitness reduction in yeast [57]. Thus, the extreme rarity of complete loss of introns in eukaryotes at least in part is likely to be due to deleterious effect of the loss of specific, functionally important introns.

## Evolutionary conservation of intron positions and routes of gene architecture evolution of eukaryotes

The realization that (nearly) all eukaryotes possess ‘genes in pieces’ but that the intron densities and size widely vary, triggered intense, ongoing discussion of possible evolutionary scenarios behind these patterns. Several mechanisms of intron evolution have been suggested including intron loss, gain, and sliding [44,58-61]. Intron loss and gain are the major phenomena in the evolution of eukaryotic gene architecture. The relative contributions of these two processes have been a matter of considerable debate and controversy. Systematic comparative analyses of exon-intron structures of orthologous genes from animals, fungi and plants have shown that approximately 25% to 30% of the intron positions are shared (that is, located in the exact same position in orthologous genes) by at least two of these three lineages of complex eukaryotes with intron-rich genomes [45,62]. The prevailing interpretation of these fundamental observations is that most, if not all, introns that occupy the same positions in orthologous genes are conserved, i.e. were already present in the equivalent position of the corresponding ancestral gene. However, the alternative view, i.e., that a substantial fraction or even most of the shared introns have been independently inserted in the same position in orthologous genes in different lines of descent, cannot be automatically dismissed (see discussion below).

The apparent conservation of many intron positions in distant eukaryotes notwithstanding, intron densities in eukaryotic genomes differ widely (see above), and the location of introns in orthologous genes does not always coincide even in closely related species [63-65]. Likely cases of intron insertion and the more common intron loss have been described (e.g., [59,63,66-82], and indications of high intron turnover rate in some eukaryotic lineages have been obtained [42]. Furthermore, parsimony and maximum likelihood (ML) reconstructions of evolution of exon-intron structure of highly conserved eukaryotic genes (see details below) suggest that both loss and gain of introns have been extensive during evolution of eukaryotic genes [45,83-88]. Together the results of these analyses indicate that the rates of intron gain and loss differ widely between eukaryotic lineages. Some eukaryotes, such as yeast *Saccharomyces cerevisiae*, seem to have lost nearly all of the ancestral introns, whereas others, e.g., nematodes, have experienced highly dynamic evolution, with both loss and acquisition of numerous introns [45,83-88]. However, intron gain is not easy to detect: comparative analysis of intron positions in orthologous genes from vertebrates revealed only a few losses but no apparent gain of introns in mammalian genes [89,90], and similar results have been obtained in an analysis of evolution of exon-intron structure of paralogous genes in several eukaryotic lineages [91]. These findings imply that intron loss dominates at short evolutionary distances, whereas bursts of intron insertion might accompany major evolutionary transitions. However, intron gain could be an ongoing process in nematodes: Coghlan and Wolfe [66] identified 81 introns in *Caenorhabditis elegans* and 41 introns in *C. briggsae* that appear to have been recently inserted. However, the validity of these recent intron gains has been questioned as it has been shown that after including additional genomes in the analysis many of the reported intron gains could be parsimoniously explained by intron loss [92]. A high rate of recent intron gain has been reported for paralogous gene pairs in *Arabidopsis thaliana* that were duplicated simultaneously 20-60 million years via tetraploidization [93]. A low rate of recent intron gains has been identified in plastid-derived genes in plants [94]. Similarly, spliceosomal introns have been detected in some genes horizontally transferred from bacteria to bdelloid rotifers [95]. Probably, the most striking known case of apparent recent intron gains has been found in populations of *Daphnia pulex* endemic to Oregon where two polymorphic introns have been identified [70]. These new introns do not have an obvious source and are not represented in any studied *D. pulex* populations outside Oregon, other species of Daphnia or any other organism for which sequence data are available. Furthermore, the new introns are both found in the same gene that encodes a Rab GTPase (rab4), and are inserted within one base pair from each other. These findings put into doubt the rarity of intron gain considering that two intron gain events have been discovered in an initial survey of only 6 nuclear loci in 36 Daphnia individuals [70]. This result was further supported by the analysis of 24 discordant intron/exon boundaries between the whole-genome sequences of two *Daphnia pulex* isolates. Sequencing of intron presence/absence loci across a collection of *D. pulex* isolates and outgroup *Daphnia* species has shown that most polymorphisms result from recent gains, with parallel gains often occurring at the same location in independent allelic lineages [96].

**Figure 3:**
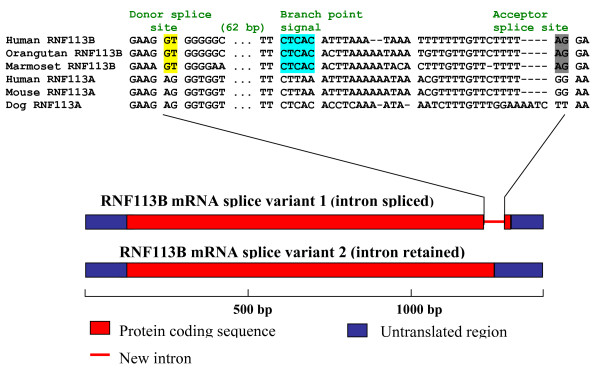
**An example of a recent intron acquisition in a retrotransposon-derived gene: structure of two splice variants of RNF113B. **The new intron of RNF113B is not a *de novo* insertion but rather a derivative of exonic sequences (this intron contains 59 nucleotides from the former coding sequence and 46 nucleotides from the 3’ UTR). A partial alignment of three RNF113B sequences and three RNF113A sequences is shown above the spliced RNF113B isoform. The donor splice site is marked in yellow, the predicted branch point signal is marked in blue, and the acceptor splice site is marked in gray. The data is from [101].

The great majority of studies aimed at reconstruction of evolution of gene architecture in eukaryotes have focused on introns in conserved portions of protein-coding regions. For example, the conclusion that there was no appreciable intron gain in mammals [89] is based on this type of data. However, evolution of poorly conserved segments of protein-coding sequences, untranslated regions of protein-coding genes, alternatively spliced regions, and genes originated from transposable elements appears to be much faster and more dynamic, with numerous intron gains in mammals [97-101]. A case of such intron acquisition has been reported for the RNF113B retrogene that encodes a RING finger protein (a predicted E3 subunit of ubiquitin ligase of unknown specificity) and is present in the genomes of all primates (Figure 3) [101]. This primate-specific gene underwent rapid evolution that included an intron gain. The presence of the intron is supported by several human mRNA sequences and comparisons with multiple primate genomes (marmoset, macaque, orangutan, and chimpanzee). Sequence alignment analysis shows that the intron of RNF113B is not a *de novo* insertion but rather a derivative of an exonic sequence (a tandem mutation AG > GT generated the donor site). The new intron contains 59 nucleotides from former coding sequence and 46 nucleotides from the 3’ UTR. This finding was further supported by sequencing of the human RNF113B cDNAs which revealed two alternative RNF113B isoforms (Figure 3) [101]. In general, due to the lack of evolutionary conservation in such genes and gene regions, reconstruction of intron gain and loss events in their evolution is difficult and sometimes inaccurate (especially without experimental verification). Accordingly, evolutionary studies tend to concentrate on highly conserved genes. Thus, the conclusions on intron stasis in some groups of eukaryotes, such as mammals, in part appear to stem from sampling biases whereas the overall intron turnover might be much more extensive than is currently appreciated. 

**Figure 4:**
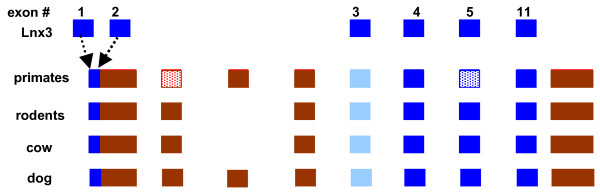
**The Xist gene evolved from a protein-coding gene and a set of transposable elements. **Blue boxes indicate exons originating from Lnx3; red boxes indicate exons originating from transposable elements; dashed boxes indicate remnants of protein-coding exons. The data is from [105].

The same problem pertains to non-coding RNA genes. For example, mammalian genomes contain numerous (> 10,000) genes for long non-coding RNAs (lncRNAs) that encompass numerous introns [102]. In a recent detailed study, over 8,000 lncRNA genes have been identified, with a mean intron density of ~1.9 per kilobase, and extensive alternative splicing of these non-coding RNAs has been detected, with ~2.3 RNA isoforms per gene [103]. One of the best studied lncRNAs is Xist which is involved in X-chromosome inactivation in females of eutherian mammals [104]. The Xist RNA appears to have evolved as a result of pseudogenization of the Lnx3 protein-coding gene in early eutherians followed by integration of mobile elements [105]. Analysis of Xist in several mammalian species revealed an overall conservation of the Xist gene structure (Figure 4). Four of the 10 Xist exons found in eutherians show significant sequence similarity to exons of the Lnx3 gene (Figure 4) whereas the remaining 6 Xist exons are similar to different transposable elements. Thus, some Xist introns were inherited from the Lnx3 gene but some appear to have been gained in the course of evolution of the Xist gene [105]. Comparative analysis of >3,000 mouse lncRNA genes suggested that conservation of the exon/intron structure might be a general lncRNA property [106]. It was found that 65% and 40% of mouse lncRNA |GT-AG| splice sites are conserved in human and rat, respectively. These numbers are significantly greater than the number of conserved intronic GT and AG dinucleotides that are not involved in splicing indicating evolutionary conservation of splice signals in lncRNAs [106]. Detailed reconstruction of the origin and evolution of introns in lncRNAs awaits further comparative genomic studies. 

The distributions of intron positions over the length of coding regions differ substantially between eukaryotic taxa. In intron-poor genes of single-cell eukaryotes, introns are strongly over-represented in the 5’-portions whereas in intron-rich multicellular organisms, the distribution is closer to uniformity [64,65]. A mechanistic explanation for these patterns suggests that introns are preferentially lost from the 3’-portion of a gene, conceivably due to the over-representation of the respective sequences among the cDNAs that are produced by reverse transcription and are thought to mediate intron loss via homologous recombination [65,107-109]. However, a complementary, selectionist interpretation of the observed distributions of introns, to the effect that introns located in the 5’-portion of a gene are more often involved in one or more intron-mediated functions (see below), has been proposed as well [65]. Analysis of distributions of intron positions over the length of the coding region suggested that both loss and insertion of introns occurred preferentially in the 3’-regions of genes, which suggested reverse-transcription-mediated mechanisms for both processes [110]. This hypothesis appears to be compatible with the positive association that has been shown to exist between the rates of intron gain and loss in individual genes [111]. However, a more recent probabilistic analysis of intron gain and loss indicates that the mechanisms of loss and gain are most likely to be different, with reverse transcription involved only in intron loss [112].

Intron sliding (also called slippage or migration; hereinafter IS) can be defined as relocation of intron/exon boundaries over short distances (1-60 bases) in the course of evolution. Intron sliding has been postulated by advocates of the introns-early hypothesis to explain the surprising finding that the positions of apparently orthologous introns sometimes varied among lineages [60]. However, the introns-late camp maintained that IS, if it occurs at all, has contributed little to the diversity of intron positions [44,59]. The reality of IS had been debated for a long time. A Monte Carlo statistical analysis of broadly sampled data on intron positions has strongly suggested that one-base-pair IS, although a relatively rare event occurring in <5% of all introns, was a bona fide evolutionary phenomenon; in contrast, no evidence supporting intron sliding over longer distances was obtained [113]. A recent study has suggested that IS might be an important source of new introns in some lineages and proposed a simple, plausible mechanism for the emergence and fixation of IS during evolution [114]. Given the near ubiquity of alternative splicing (AS) in many groups of animals and possibly plants [48], Tarrio et al. proposed that AS could be an intermediate stage in the evolution of IS. Under this scenario, emergence of a new splicing signal adjacent to a pre-existing one results in AS but, if and when the original splicing signal subsequently deteriorates, the net result is IS [114]. The proposed route of IS evolution via AS is likely to be common in poorly conserved regions of protein-coding genes with frequent AS events (e.g. 5’- and 3’-regions of many genes) but rare in conserved portions of protein-coding genes. Comparative analysis of closely located introns among 12 *Drosophila* genomes has suggested that IS is a relatively frequent cause of novel intron positions in *Drosophila*[115]. All things considered, there is currently no doubt that IS is real and can yield new intron positions but the actual impact of IS in the evolution of eukaryotic genes will be accurately determined only when multiple sets of closely related genomes become available and rigorous methods for statistical analysis are developed.

## Evolution of splicing signals, protosplice sites, and intron phase distribution

As pointed out above, the densities of spliceosomal introns vary dramatically among eukaryotes (Figure 2), and so does the strength of splicing signals [18,45,51,116]. There is a striking correspondence between low intron density and high information content of donor splice signals across eukaryotic genomes [51]. Intron-poor genes (genomes) with strong donor sites are found in several groups of eukaryotes (e.g. fungi) that also include intron-rich genomes with weaker donor sites. Evolutionary reconstruction suggests that ancestral donor signals had low information content but that many lineages have independently underwent concomitant major intron loss and donor signal strengthening [51]. This evolutionary trend receives a straightforward explanation within the framework of the population-genetic concept of evolution of gene architecture (see below).

**Figure 5:**
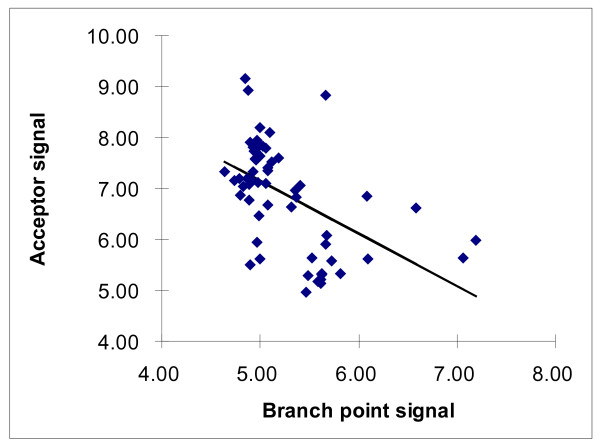
**Correlation between the strength of the branch point signal and the strength of the acceptor splice site.** The linear correlation coefficient is R = -0.52 (P = 0.000025) after exclusion of the obvious outlier *Aureococcus anophagefferens*[117]. The information content of splicing signals in 61 eukaryotic species is from [117]. Species names: *B. taurus, C. familiaris, E. caballus, H. sapiens, M. domestica, M. musculus, O. anatinus, R. norvegicus, S. scrofa, B. florida, C. intestinalis, C. savignyi, D. rerio, G. gallus, O. latipes, P. marinus, T. guttata, X. tropicalis, A. gambiae, A. mellifera, C. elegans, D. pulex, D. melanogaster, H. magnipapillata, L. gigantea, M. brevicollis, N. vectensis, S. purpuratus, T. castaneum, B. dendrobatidis, C. heterostrophus, C. neoformans, M. grisea, N. haematococca, P. chrysosporium, P. blakesleeanus, P. infestans, P. placenta, S. cerevisiae, S. commune, T. virens, A. anophagefferens, D. discoideum, D. purpureum, N. gruberi, O. lucimarinus, P. tricornutum, T. pseudonana, T. adhaerens, A. thaliana, Chlorella NC64A, C. reinhardtii, M. pusilla, Micromonas RCC299, O. sativa, P. patens, P. trichocarpa, S. moellendorffii, S. bicolor, V. vinifera, V. carteri.*

However, the acceptor splice signal shows a different trend: it is weak in most fungi, intermediate in plants and some unicellular eukaryotes, and strongest in metazoans where it gradually strengthens from nematodes to mammals [116]. This observation can be interpreted in the light of the results of a large-scale analysis of splicing signals in 61 eukaryotic species which revealed a significant negative correlation between the strength of the branch point signal and the strength of the acceptor splice site (Figure 5; R = -0.52, P = 0.000025) [117]. Although the correlation between the strength of the donor splice signal and the combined strength of the branch point signal and the acceptor splice signal was not significant (R = 0.19, P = 0.15), the positive sign of this correlation still could reflect congruent evolution of splicing signals. In general, a complex interplay exists between intron density, intron size, the strength of splice signals and the strength of splicing enhancers/silencers. For example, splice signals in long and short introns in Drosophila show only minor differences [118]. Several weak but statistically significant correlations have been observed between vertebrate intron length, splice site strength, and potential exonic splicing signals that attest to a compensatory relationship between splice sites and exonic splicing signals, depending on vertebrate intron length [119]. 

It has been proposed that the functionally important (A/C)AG||G exon sequences flanking introns are relics of recognition signals for the insertion of introns that have been dubbed protosplice sites [14,15]. Protosplice sites became an important staple of the introns-late hypothesis of intron evolution because, if intron insertion was limited to strictly defined protosplice sites, parallel gain of introns would be likely and could account for the large number of shared introns among orthologs from distant eukaryotic lineages [41,63,83]. Support for the protosplice site hypothesis has been harnessed from experiments demonstrating that elimination of the regular splice sites in actin genes resulted in activation of cryptic splice sites, most of which coincided with exon junctions in orthologous genes from other species [120]. Nevertheless, it remained unclear whether the consensus nucleotides flanking the splice junctions were remnants of the original protosplice sites or evolved convergently after intron insertion. The existence of protosplice sites was directly addressed by examining the context of introns inserted within codons which encode amino acids conserved in all eukaryotes and, accordingly, are not subject to selection for splicing efficiency. According to the parsimony principle, these codons (e.g., GGN for conserved glycines or CCN for conserved prolines) can be inferred to have been present already in the common ancestor of all extant eukaryotes, so the ancient protosplice sites (if such existed) should have survived and could be examined directly. This analysis has shown that introns, indeed, predominantly insert into and/or are preferentially fixed in specific (protosplice) sites with the consensus sequence (A/C)AG||Gt [121].

Recently, correlation between positions of cryptic splicing signals (sequences that are similar to splicing signals but normally do not function in splicing) and introns has been found: cryptic splicing signals within exons of one species frequently match the exact position of introns in orthologous genes from another species. This observation suggests that in the course of evolution many introns were inserted into cryptic splicing signals that had been in place prior to intron insertion [122]. However, this conclusion is contradicted by another observation, that recently gained introns in animal genes of the alpha-amylase were not associated with specific sequence motifs (protosplice sites) [123]. In the same gene family, old introns were embedded within strong protosplice motifs which were found to be much weaker in homologous genes lacking the intron in the given position [123]. These findings are consistent with the hypothesis that sites of *de novo* intron insertion are effectively random and that selection drives the emergence of protosplice-like sequences following intron insertion. The presence of much stronger protosplice sites around old introns compared to young introns [123] seems to suggest that evolution of protosplice sites subsequent to intron insertion is a slow process [123,124].

The hypothesis that selection acts on the exonic parts of splice signals was supported by comparison of the nucleotide sequences around the splice junctions that flank old (shared by two or more major lineages of eukaryotes) compared with new (lineage-specific) introns in eukaryotic genes. The distributions of information content between the intronic and exonic parts of the splices signals have been found to be substantially different in old and new introns [125]. Old introns have lower information content in the exonic regions adjacent to the splice sites than new introns but, conversely, have higher information content in the intron itself. These findings imply that introns insert into protosplice sites but during the evolution of an intron after insertion, the splice signal shifts from the flanking exonic regions to the ends of the intron itself. Accumulation of information inside the intron during evolution is best compatible with the view that new introns, largely, emerge *de novo* and not via propagation and migration of pre-existing introns [125].

The contradictory findings on the protosplice site involvement versus the evolution of these motifs after intron gain (in which case ‘protosplice site’ becomes a misnomer) might reflect objectively existing differences in the evolution of the gene architectures among gene families, in particular between highly conserved and more dynamic families. The definitive assessment of the validity of the protosplice site hypothesis requires further, comprehensive comparative genomic studies.

Introns occur in three phases (0, 1, and 2) which are defined as the position of the intron within or between codons: introns of phase 0, 1, and 2 are located, respectively, between two codons, after the first position in a codon, and after the second position. In (nearly) all analyzed genomes, there is a significant excess of phase 0 introns over those in the other two phases [125-130]. The only known remarkable exception is the rapidly evolving tunicate *Oikopleura* that shows a uniform distribution of introns among the three phases [131].

**Figure 6:**
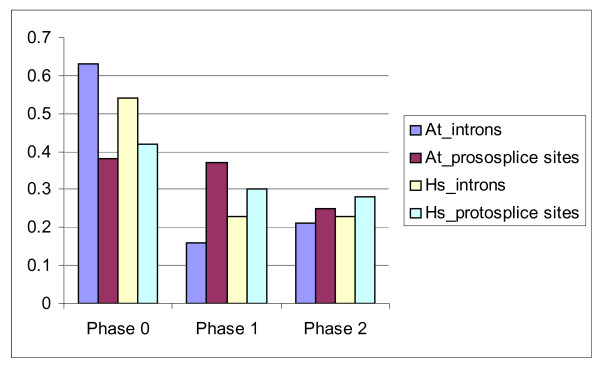
**Fractions of protosplice sites and actual introns in the three phases. **Species abbreviations: (At) green plant *Arabidopsis thaliana*, (Hs) human *Homo sapiens*. An excess of protosplice sites in phase 0 is noticeable, however the ‘protosplice site’ hypothesis, which posits that introns are randomly inserted into protosplice sites, is unable to fully explain the observed over-representation of phase 0 introns. The data is from [125,132].

An excess of protosplice sites in phase 0 was noticeable in some species (Figure 6) [132], however the protosplice site model cannot fully explain the observed over-representation of phase 0 introns under the assumption that introns randomly insert into protosplice sites (Figure 6) [125,127,128]. Furthermore, it has been shown that phase 0 introns were, on average, located in more highly conserved portions of genes than phase 1 and 2 introns [45]. This observation suggests that phase 1 and phase 2 introns experience a greater deleterious-mutation-driven loss and could reconcile the observed phase distribution with the protosplice site hypothesis [125,127,128,130]. 

## Conservation versus parallel gains of introns

As discussed above, comparative analyses revealed numerous introns that occupy the same position in orthologous genes from distant species [45,62]. In particular, orthologous genes from humans and the green plant *A. thaliana* share ~25% intron positions [45]. The straightforward interpretation of these observations is that the shared introns were inherited from the common ancestor of the respective species whereas lineage-specific introns were inserted into genes at later stages of evolution [45,62]. Under this premise, parsimonious reconstructions indicate that even early eukaryotes already had a relatively high intron density, perhaps, comparable (at least within an order of magnitude) to that in modern plant and animal genes. However, the inference that shared intron positions reflect evolutionary conservation is challenged by the potential non-randomness of intron insertion: introns are inserted or fixed mostly in distinct protosplice sites as discussed in the preceding section. In principle, if there were few protosplice sites in each gene, the presence of introns in the same positions of orthologous genes in distantly related species could be completely or at least to a large extent explained by parallel gains. At least two cases of apparent parallel gain of introns in orthologous genes from plants and animals have been reported [133,134]. Moreover, probabilistic modeling of intron evolution discussed above suggested that many, if not most, introns shared by phylogenetically distant species were likely to originate by parallel gain of introns in protosplice sites [83]. The implication is that intron distribution in extant organisms is largely determined by relatively recent insertions and cannot be used to infer exon-intron structure of ancestral genes. However, the dataset of 10 gene superfamilies by Qiu and co-workers [83] contained numerous ancient duplications combined with frequent lineage-specific losses of genes, because of which analysis of intron conservation and intron gains is likely to be confounded by problems of phylogenetic reconstructions.

The extent of independent insertion of introns in the same sites (parallel gain) in orthologous genes from phylogenetically distant eukaryotes was assessed within the framework of the protosplice site model [132]. It was shown that protosplice sites are no more conserved during evolution of eukaryotic gene sequences than random sites. Simulation of intron insertion into protosplice sites with the observed protosplice site frequencies and intron densities has shown that parallel gain could account for only 5 to 10% of shared intron positions in distantly related species [132]. The results of this simulation suggest that the presence of numerous introns in the same positions in orthologous genes from diverse eukaryotes, such as animals, fungi, and plants, reflects mostly *bona fide* evolutionary conservation [132].

Analysis of intron gain and loss rates over branches of the phylogenetic tree for 19 eukaryotic species allowed for the estimation of the probability of parallel gain for each intron position that is shared by more than one species [111]. The resulting estimates indicated that parallel gain, on average, accounts for only ~8% of the shared intron positions, in agreement with the simulation results discussed above [111]. However, the distribution of parallel gains over the phylogenetic tree of eukaryotes is highly non-uniform. There are almost no parallel gains in closely related lineages, whereas for distant lineages, such as animals and plants, parallel gains could contribute up to 20% of the shared intron positions. Taken together, the results of these analyses indicate that, although parallel gain of introns is non-negligible, the substantial majority of introns that occupy the same positions in orthologous genes from distantly related eukaryotes are ancestral including many inherited from LECA [111].

## Reconstruction of evolution of exon-intron structure of eukaryote genes

The patterns of conservation and variation of intron positions in orthologous and paralogous genes can be employed to reconstruct evolutionary scenarios for the exon-intron structure of eukaryotic genes using evolutionary parsimony or maximum likelihood approaches. Once multiple eukaryotic genomes have been sequenced, such genome-wide evolutionary reconstruction has become realistic. The comparative data on intron positions can be naturally represented as a matrix of intron presence/absence (usually encoded as 1/0), and to these matrices, various reconstruction methods can be applied. The first of such studies employed orthologous gene sets from 8 eukaryotic species and took the most straightforward approach to evolutionary reconstruction by applying the parsimony principle in the specific form of Dollo parsimony [45]. Given a species tree topology and a pattern of intron presence/absence, the Dollo algorithm constructs the most parsimonious (simplest) scenario for the evolution of gene structure, i.e. the distribution of inferred intron gain and loss events over the tree branches. The main underlying assumption is that a character (intron in a given position) once lost cannot be regained whereas as many parallel intron losses in different branches of the tree are allowed as needed to account for the observed pattern of states. By analyzing more than 7,000 intron positions in highly conserved genes of eukaryotes, the Dollo parsimony approach produced an evolutionary scenario under which the common ancestor of modern eukaryotes possessed intron-rich genes, with intron density only a few fold lower than that in most intron-rich modern forms (vertebrates and plants). Massive intron losses were inferred for several groups, especially yeasts, nematodes and insects, whereas in vertebrates and plants intron gain was inferred to be the main evolutionary trend [45].

The parsimony approach is obviously oversimplified given that all lineage-specific introns are automatically treated as newly gained, with the possibility that some of these introns could be ancestral, having been lost in multiple lines of descent. Furthermore, parsimony does not provide confidence estimates for the estimates of ancestral states. These limitations of parsimony potentially could grossly distort the results of evolutionary reconstruction, especially if the number of analyzed species is small. Probabilistic approaches such as maximum likelihood models can address these problems, at least in principle. However, the first two statistical studies into intron evolution produced opposite results: Qiu et al. concluded that ancient introns (if they ever existed) have not survived in extant genes [83] whereas Roy and Gilbert came to the conclusion that the great majority of introns, even lineage-specific ones, were ancient [84]. The first conclusion implies that intron gain was dominant over intron loss in the evolution of eukaryotic genes, whereas the second one suggests that intron loss is the principal evolutionary process. This major discrepancy between the results of the two studies has indicated that optimal parameters for maximum likelihood modeling of intron evolution remained to be determined [135].

The next generation of increasingly sophisticated ML reconstructions of intron gain and loss during eukaryotic evolution suggested that the protein-coding genes of ancient eukaryotic ancestors, including the Last Eukaryotic Common Ancestor (LECA), already possessed intron density comparable to that found in modern, moderately intron-rich genomes [85-88,136]. Accordingly, the history of eukaryotic genes, with respect to the dynamics of introns, appears to be to a large extent dominated by losses, perhaps punctuated by a few episodes of major gain [87,88,91,137]. This conclusion is based on analyses xof considerably larger data sets than those used in earlier studies; for example, Carmel and co-workers [87] analyzed 391 sets of orthologous genes from 19 eukaryotic species. This extended data set not only allowed for a more definitive reconstruction of the gene structure evolution, but also permitted zooming in on specific portions of the eukaryotic tree [87]. A comprehensive probabilistic model of intron evolution was developed that incorporated heterogeneity of intron gain and intron loss rates with respect to both lineages and genes as well as variability among sites within a gene [87]. It was demonstrated that ancestral eukaryotic forms were intron-rich and that evolution of eukaryotic genes involved numerous gains and losses of introns, with losses being somewhat more common. Three distinct modalities of intron gain and loss during eukaryotic evolution were identified. The ‘balanced’ mode appears to operate in all eukaryotic lineages, and is characterized by proportional intron gain and loss rates [87]. In addition to this, apparently universal process, many lineages exhibit elevated loss rate, whereas a few others exhibit elevated gain rate. Moreover, the rates of intron gain and loss are highly non-uniform over the time course of the evolution of eukaryotes: both rates seem to have been decreasing with time over the last several hundred million years. The decrease in gains was faster than the decrease in losses, resulting in many lineages with very limited intron gain over the last several hundred million years [87].

**Figure 7:**
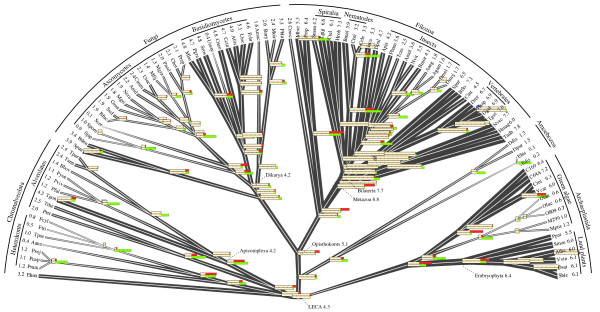
**Reconstruction of intron gains and losses in the evolution of eukaryotes and intron density in ancestral eukaryote forms.** The data is from [53]. Branch widths are proportional to intron density which is shown next to terminal taxa and some deep ancestors, in units of the introns count per 1 kbp coding sequence. Human (Hsap) is marked by a blue dot. Horizontal bars show ancestral (top) and current (bottom) intron content; gain and loss (in the lineage from the respective ancestor) are shown by red and green, respectively. The bars are aligned so that the pale yellow part shows the retained introns from the ancestor. Species names and abbreviations: *Aureococcus anophagefferens* (Aano), *Aedes aegypti* (Aaeg), *Agaricusbisporus* (Abis), *Anopheles gambiae* (Agam), *Allomyces macrogynus* (Amac), *Apis mellifera* (Amel), *Aspergillus nidulans* (Anid), *Acyrthosiphon pisum* (Apis), *Arabidopsis thaliana (*Atha), *Babesia bovis* (Bbov), *Batrachochytrium dendrobatidis* (Bden), *Branchiostoma floridae* (Bflo), *Botryotinia fuckeliana* (Bfuc), *Brugia malayi* (Bmal), *Bombyx mori* (Bmor), *Coccomyxa sp*. C-169 (C169), *Chlorella sp*. NC64a (C64a*), Caenorhabditis briggsae* (Cbri), *Caenorhabditis elegans* (Cele), *Coprinopsis cinerea okayama* (Ccin), *Cochliobolus heterostrophus* C5 (Chet), *Coccidioides immitis* (Cimm), *Ciona intestinalis* (Cint*), Cryptococcus neoformans var. neoformans* (Cneo*), Chlamydomonas reinhardtii* (Crei), *Capitella teleta* (Ctel), *Capsaspora owczarzaki* (Cowc), *Dictyostelium discoideum* (Ddis), *Dictyostelium purpureum* (Dpur), *Drosophila melanogaster* (Dmel), *Drosophila mojavenis* (Dmoj), *Daphnia pulex* (Dpul), *Danio rerio* (Drer), *Entamoeba dispar* (Edis), *Entamoeba histolytica* (Ehis), *Emiliania huxleyi* (Ehux), *Fragilariopsis cylindrus* (Fcyl), *Phanerochaete chrysosporium* (Fchr), *Phaeodactylum tricornutum* (Ftri), *Gallus gallus* (Ggal), *Gibberella zeae* (Gzea), *Hydra magnipapillata* (Hmag), *Helobdella robusta* (Hrob), *Homo sapiens* (Hsap), *Ixodes scapularis* (Isca), *Laccaria bicolor* (Lbic), *Lottia gigantea* (Lgig), *Micromonas sp*. RCC299 (M299), *Monosiga brevicollis* (Mbre), *Mucor circinelloides* (Mcir*), Mycosphaerella fijiensis* (Mfij), *Mycosphaerella graminicola* (Mgra), *Magnaporthe grisea* (Mgri), *Melampsora laricis-populina* (Mlar), *Micromonas pusilla* (Mpus), *Neurospora crassa* (Ncra), *Nematostella vectensis* (Nvec), *Nasonia vitripennis* (Nvit), *Ostreococcus sp*. RCC809 (O809), *Ostreococcus lucimarinus* (Oluc), *Oryza sativa japonica* (Osat), *Ostreococcus taurii* (Otau), *Phytophthora capsici* (Pcap), *Plasmodium falciparum* (Pfal), *Puccinia graminis* (Pgra), *Pediculus humanus* (Phum), *Phaeosphaeria nodorum* (Pnod), *Physcomitrella patens subsp. patens* (Ppat), *Phytophthora ramorum* (Pram), *Pyrenophora tritici-repentis* (Prep), *Proterospongia sp*. (Prsp), *Phytophthora sojae* (Psoj), *Paramecium tetraurelia* (Ptet), *Plasmodium vivax* (Pviv), *Plasmodium yoelii yoelii* (Pyoe), *Rhizopus oryzae* (Rory*), Sorghum bicolor* (Sbic), *Saccharomyces cerevisiae* (Scer), *Schizosaccharomyces japonicus* (Sjap), *Schistosoma mansoni* (Sman), *Selaginella moellendorffii* (Smoe), *Schizosaccharomyces pombe* (Spom), *Spizellomyces punctatus* (Spun), *Strongylocentrotus purpuratus* (Spur), *Sporobolomyces roseus* (Sros), *Sclerotinia sclerotiorum* (Sscl), *Trichoplax adhaerens* (Tadh), *Theileria annulata* (Tann), *Tribolium castaneum* (Tcas), *Toxoplasma gondii* (Tgon), *Taenopygia guttata* (Tgut), *Theileria parvum* (Tpar), *Thalassiosira pseudonana* (Tpse), *Tetrahymena thermophila* (Tthe), *Ustilago maydis* (Umay), *Uncinocarpus reesii* (Uree), *Volvox carteri* (Vcar), *Vitis vinifera* (Vvin).

Figure 7 illustrates the latest reconstruction of intron gain and loss for 99 eukaryotic species that was performed using a Markov Chain Monte Carlo (MCMC) approach [53]. In this, so far the most extensive study, the Malin software package [138] was used to infer ancestral states from a matrix of shared introns which comprised 8403 intron presence-absence profiles from 245 sets of orthologous genes. The MCMC method infers ancestral intron density by using a probabilistic intron gain-loss model, taking into account rate heterogeneity across lineages and across sites within genes. This reconstruction provides a thorough view of the evolution of gene structure in three eukaryotic supergroups and reveals several general trends (Figure 7) [53]. Most lineages show net intron loss that can be substantial as in alveolates, some lineages of fungi, green algae or insects, or offset by concomitant intron gains as in land plants, most animal lineages, and some fungi. Massive intron gains were inferred only for several deep branches, most conspicuously the stem of the Metazoa, and to a lesser extent, the stems of Mamiellales (a branch of green algae), Viridiplantae, Opisthokonta, and Metazoa together with Choanoflagellata (Figure 7). These findings vindicate, on a much larger data set and with greater confidence, the previous conclusions that compared to the common and substantial intron loss, extensive intron gain was rare during the evolution of eukaryotes. Episodes of substantial intron gain seem to coincide with the emergence of major new groups of organisms with novel biological characteristics such as the Metazoa (Figure 7) [53]. Several previous studies, performed on much smaller data sets and with less robust reconstruction methods, have suggested that at least some eukaryotic ancestral forms could have possessed intron-rich genes [84,85,136], and observations on gene structures in primitive animals such as the sea anemone *Nematostella*[139] and the flatworm *Platynereis*[140] were compatible with these inferences. A particularly striking conclusion has been reached in the reconstruction of the evolution of gene architecture in Chromalveolata: although all sequenced genomes in this supergroup of eukaryotes are intron-poor, intron-rich last common ancestors have been inferred for Chromalveolata and particularly Alveolata [141]. Clearly, the reconstruction led to this conclusion only because, although very few intron positions are conserved among the intron–poor orthologous genes of different chromalveolates, many of these genes share a large fraction of intron positions with intron-rich orthologs from plants and/or animals. 

The latest MCMC reconstruction reinforced these conclusions by inferring high intron densities for the ancestors of each major group of eukaryotes within each of the three supergroups (Figure 7) [53]. The implication is that, whenever an extant eukaryotic genome shows a low intron density, this intron-poor state is a result of extensive, lineage-specific intron loss. Remarkably, so many intron positions are shared between eukaryotes that, with the large and apparently representative set of compared genomes, Dollo parsimony reconstruction infers similarly intron-rich ancestral genomes as the MCMC and maximum likelihood methods [53]. The results of this reconstruction indicate in particular that the entire line of descent from LECA to mammals was a continuous intron-rich state (Figure 7) that would provide for uninterrupted evolution of the growing repertoire of functional alternative spliced forms (see below). The unprecedented intron gain at the onset of animal evolution (Figure 7) could further contribute to the expansion of alternative forms. This spurt of intron gain conceivably resulted from a combination of a population bottleneck that led to weak purifying selection with increased transposon activity (see below).

## Evolution of exon-intron structure in paralogous gene families

The reconstructions of the evolution of gene architecture in eukaryotes were performed on sets of orthologous genes with a single representative (or a single most conserved representative) in each of the analyzed genomes. Obviously, this type of reconstruction reflects only one facet of evolution of gene structure given that all eukaryotic genomes encompass numerous families of paralogous genes with broad distributions of the number of members. Reconstruction of parsimonious scenarios of gene structure evolution in paralogous gene families in animals, plants and malaria parasites revealed numerous apparent gains and losses of introns [91,142]. In all analyzed lineages, the number of acquired new introns was substantially greater than the number of lost ancestral introns. This trend held even for lineages in which vertical evolution of genes involved many more intron losses than gains, suggesting that gene duplication boosts intron insertion. However, dating gene duplications and the associated intron gains and losses based on the molecular clock assumption showed that very few, if any, introns were gained during the last approximately 100 million years of animal and plant evolution, in agreement with previous conclusions reached through analysis of orthologous gene sets. These results are generally compatible with the emerging notion of intensive insertion and loss of introns during transitional epochs in contrast to the relative quiet (stasis) of the intervening evolutionary spans [91,137,143]. The prevalence of intron gain over intron loss in evolving families of paralogs remains a somewhat controversial issue. It has been suggested that the Dollo parsimony approach used by Babenko and co-workers [91] could significantly underestimate the rate of intron losses [144]. However, even should that be the case, the independently estimated number of intron gains in the same data set that was used in the original work of Babenko and coworkers [91] still exceeded the number of intron losses [144]. Furthermore, numerous anecdotal observations (e.g., [93,145-147]) have suggested that at least some paralagous gene families have gained more introns than they have lost.

**Figure 8:**
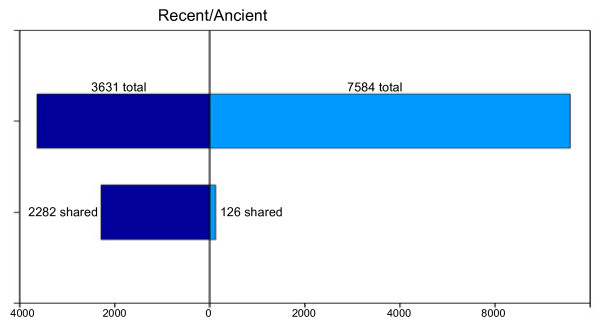
**Conservation of intron positions in ancient and recent eukaryotic paralogs.** Conservation of introns was assessed by analysis of multiple alignments of paralogous sequences from 6 species (*H. sapiens, C. elegans, D. melanogaster, S. pombe, S. cerevisiae, A. thaliana*). An intron position was considered to be conserved if it was shared by any pair of paralogs [148].

**Figure 9:**
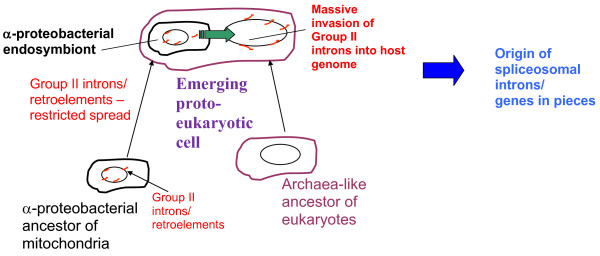
**A hypothetical scenario of early history of spliceosomal introns. **The scheme shows the inferred sequence of events from putative ancestors of eukaryotes to the origin of spliceosomal introns from group II introns invading the host genome upon mitochondrial endosymbiosis [46].

In contrast, comparison of the exon–intron structures of ancient eukaryotic paralogs reveals the absence of conserved intron positions in these genes (Figure 8) [148]. This is in contrast to the conservation of intron positions in orthologous genes from even the most evolutionarily distant eukaryotes and in more recent paralogs (Figure 8) [91]. The lack of conserved intron positions in ancient eukaryotic paralogs probably reflects the origin of these genes during the earliest phase of eukaryotic evolution that was characterized by concomitant invasion of genes by group II self-splicing elements (which were to become spliceosomal introns subsequently; see below) (Figure 9) and extensive duplication of genes [148,149]. Similar estimates were obtained for parallel intron gains in ‘pseudo-paralogous’ genes encoding cytosolic and mitochondrial ribosomal proteins that by definition have acquired their intron independently: approximately 2.3% of the intron positions were found in homologous positions [150]. The lack of conserved introns in ancient eukaryotic paralogs [148,150] is consistent with the results of an earlier analysis of intron distribution in 20 most ancient (duplicated before the divergence of bacteria and archaea) paralogous families which appear to have accumulated introns independently [151]. Along with other lines of evidence, these observations do not seem to be compatible with the introns early hypothesis. 

## Evolution of exon-intron structure in connection with other features of eukaryote genes

The combined advances of comparative genomics and systems biology provide means to characterize genes by many features, for example expression level and connectivity in protein-protein interaction or regulatory networks. Various significant correlations have been demonstrated to exist between these variables; in particular, one of the most prominent, recurrent links is that the sequence of highly expressed genes tends, on average, to be more conserved [152-154]. Connections between various features of introns and other characteristics of genes also have emerged. Here, we discuss the links between two key features of introns, the rates of gain and loss and intron length, and other aspects of gene evolution, expression and function.

Probabilistic evolutionary reconstruction of gene structure yields gene-specific rates of intron gain and loss and thus provides for analysis of possible relationships between these rates and other characteristics of the respective genes [87]. It has been shown that intron gain rate was negatively and significantly correlated with the sequence evolutionary rate; conversely, intron loss rate was positively and significantly correlated with the rate of sequence evolution. Thus, perhaps somewhat counter-intuitively, highly conserved genes appear to accumulate introns in the course of evolution, even if slowly. Also significant, although of a lesser magnitude, was the positive correlation between gene expression level and intron gain rate and the converse negative of expression with intron loss rate. This finding suggests that introns might contribute to optimal gene expression [155] although this effect is confounded by the stronger connection between expression and evolution rate.

Although expression may be enhanced by the mere presence of multiple introns in a gene, highly expressed gene in human and *Drosophila* have, on average, shorter introns than genes expressed at a lower level [156]. This finding has been subsequently validated and expanded by several independent research groups on other model eukaryotes, for exon lengths as well, and for a variety of methods used to measure expression level [157-165]. Two competing (although not necessarily mutually exclusive) hypotheses have been proposed to explain the apparent link between gene compactness and expression. The selection hypothesis holds that evolution of highly expressed genes is driven by selection for minimization of the time of transcription and/or energy expenditure resulting in shrinking of these genes, especially introns [156]. The alternative view, known as the genomic design hypothesis, holds that genes that are expressed under tight tissue- and developmental stage-dependent control require complex regulation and therefore need long introns to accommodate additional regulatory elements. Under the genomic design view, due to the positive association between the breadth and rate of gene expression, genes that are constitutively expressed at a high level and do not require complex regulation possess shorter introns [160].

**Figure 10:**
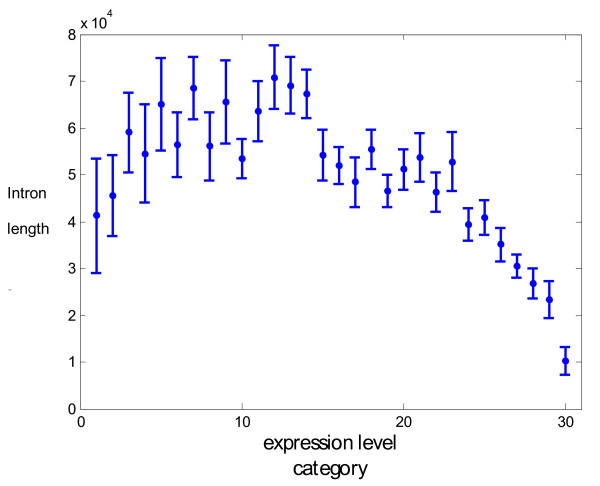
**Total intron length as a function of expression level category. **Intron length is measured in nucleotides. Expression levels are binned into 30 categories, with higher categories matching higher expression levels, as described previously [167]. Each point is the mean value for all genes in the given expression category, and the error bar indicates the standard deviation of the mean.

Surprisingly, however, the opposite trend has been reported to exist in plants, with highly expressed genes containing longer introns [166]. This discrepancy was resolved by examining the relationship between gene length and expression level at a finer resolution: the relationship between intron length (as well as other measures of gene compactness such as the length of exons or entire genes) and expression level is universal across all eukaryotes (for which sufficient amount of data on expression was available) but is non-monotonic [167]. Genes that are highly expressed indeed tend to have shorter introns but genes expressed at low to medium levels show a positive correlation between intron length and expression; hence a roughly bell-shaped dependency between expression level and intron length (Figure 10) [167]. The phenomena that underlie this non-monotonic dependency are not quite clear but might involve competition between two opposing trends. Selective pressure to maximize expression rate and minimize energy expenditure could be dominant in highly expressed genes as originally suggested [156]. In contrast, requirement for more complex regulation in moderately expressed genes that gain additional functions with increased expression might result in the positive correlation between intron length and expression [167]. 

## A population-genetic perspective on evolution of introns and eukaryotic gene architecture

The question famously posed by Walter Gilbert in the seminal note on the origin of splicing [1] - *Why Genes in Pieces?* - certainly remains pertinent to this day. To further sharpen the question: Why are some genomes, in particular those of multicellular eukaryotes (plants and animals), intron-rich whereas others, i.e. those of the great majority of unicellular eukaryotes, are intron-poor? In principle, accumulation of introns in genes of multicellular organisms could be considered as an adaptation that ensures evolution of organismal complexity, especially via AS. This is indeed the position taken by the proponents of the genome design hypothesis discussed in the preceding section. However, a simpler explanation that appears to be better compatible with the data has been proposed by Lynch as part of the non-adaptive theory for the evolution of complexity [42,49,50,168,169]. A simple estimate based on the number of nucleotide sites required for accurate intron excision during splicing (that is, the donor and acceptor sites and the branching point motif) shows that the power of purifying selection is sufficient to eliminate the majority of introns only in populations with a large effective population size (*Ne*) such as found in many unicellular eukaryotes (*Ne* ~ 10^7^ - 10^8^) [50,170] but not in the relatively small populations of vascular plants and vertebrates (Ne ~ 10^5^-10^6^ and 10^4^-10^5^, respectively) [50,170,171]. Numerical simulations based on this estimate reveal a phase transition-like shift from intron-rich to intron-poor genomes [50,168,169] which roughly matches the observed distribution of intron densities (see Figure 2).

This non-adaptive population genetic perspective on the evolution of introns and eukaryotic gene architecture is compatible with the results of empirical reconstruction according to which the general (perhaps counter-intuitive) trend is evolution of intron-poor genomes in multiple lineages from intron-rich ancestors (see Figure 2). This evolutionary trend appears to be a form of ‘genomic streamlining’ occurring in evolutionarily successful lineages that reach high effective population sizes which prevent effects of genetic drift and eliminate even slightly deleterious features such as introns. Conversely, the apparent bursts of intron gain linked to the origin of major groups of eukaryotes such as the Metazoa would coincide with population bottlenecks which are typical of such transitional epochs [42,49,50,172]. The non-adaptive population genetic concept is also compatible with the finding that intron-rich organisms possess much weaker donor splice signal than intron-poor organisms: the pressure of purifying selection in intron-rich lineages is insufficient to strictly maintain the consensus nucleotides at the donor sites [51]. A more direct analysis that compared the rates of consensus-to-variant and variant-to-consensus substitutions in the donor sites of three intron-rich lineages supported the existence of purifying selection against variants that, however, is too weak to maintain the consensus in most of the introns [52].

A major consequence of the inability of purifying selection in small populations to eliminate introns or to maintain strong donor splice signals is the accumulation of aberrant splice variants. Such error-prone splicing could eventually give rise to functional alternative splicing. Notably, the latest scenario of intron gain and loss in widespread eukaryotic genes includes only intron-rich intermediates on the path of evolution from the LECA to mammals (see above; Figure 7), with the implication that this line of descent never went through a stage of strong purifying selection allowing continuous evolution of alternative splice variants [53].

Although the non-adaptive population genetic theory appears to be the best available conceptual framework for the evolution of eukaryotic gene architecture, splicing and introns, at least two notable problems remain outstanding. First, it is unclear why the acceptor splice signal does not follow the same trend as the donor site and is stronger in intron-rich multicellular eukaryotes than it is in intron-poor unicellular forms although the observed positive correlation between the strength of the donor splicing signal and the combined strength of the branch point signal + acceptor splice signal [117] might explain this incongruence. Second, the preservation of at least a few introns even in the most intron-poor organisms remains enigmatic because at face value the non-adaptive scenario would predict complete loss of introns and accordingly the spliceosome in multiple lineages.

## Evolution of alternative splicing in coding and non-coding regions of eukaryote genes

In multicellular organisms, particularly animals, AS is a major mechanism for regulating gene expression and function [173-176]. Large-scale studies based on mapping of expressed sequence data on genomic sequences and RNAseq surveys have shown that more than 90% of human and over 40% of *Arabidopsis thaliana* and rice genes are capable of producing multiple diverse mRNA molecules through alternative splicing [177-183].

Alternative splicing has been identified in many eukaryotic groups; however, it remains unclear whether frequent alternative splicing emerged early in eukaryotic evolution [176,184] because ancestral splice signals were weak and failed to provide for highly accurate splicing, or has evolved more recently and independently in multiple lineages via mutation of strong ancestral splice signal in multi-intron genes [33]. As pointed out in the previous section, the non-adaptive population genetic model that is in excellent agreement with the empirical reconstructions of eukaryote gene architecture evolution implies that AS evolved already at the earliest stages of eukaryote evolution through accumulation of aberrant splice variants under conditions of weak purifying selection. A further implication of this scenario is that initially all alternative transcripts were non-functional whereas functional AS evolved gradually and independently in multiple lineages, primarily those that have never gone through population bottlenecks leading to extensive loss of introns and tightening of splice signals.

The impact of alternative splicing on protein function has been studied in great detail and is generally recognized as a major source of protein diversity that greatly expands the repertoire of protein function [173-175]. A systematic comparison of 9 animal genomes from nematodes to mammals revealed that intron-flanking domains expanded faster than other protein domains [185]. Intriguingly, such mobile domains exhibited a strong preference for phase 1 introns [185-188] in contrast to the general excess of phase 0 among introns (Figure 6). This finding suggests that evolution of introns flanking mobile domains is fundamentally different from the evolution of introns in conserved portions of genes but the nature of these differences remains to be elucidated [185,187,188].

Evolutionary conservation of alternative splicing is a controversial matter. Only limited conservation of alternatively spliced (cassette) exons within mammals and within dipterans has been detected [189-193]. However, a strikingly different pattern has been reported for *Caenorhabditis* nematodes: more than 92% of cassette exons found in *C. elegans* are conserved in *C. briggsae* and/or *C. remanei*[194], The differences in conservation between lineages might reflect differences in the fractions of functional alternative transcripts but possibly also differences in intron length and the strength of splicing signals [194].

Evolution of alternative splicing has been also analyzed in the context of splicing signals [195]. The GT dinucleotide in the first two intron positions is the most conserved element of the U2 donor splice signal. However, in a small fraction of donor signals (<1%), GT is replaced by GC. A substantial enrichment of GC in donor signals of alternatively spliced genes has been observed in human, nematode and Arabidopsis, suggesting that GC signals are important for regulation of alternative splicing [196-198]. Parsimony analysis was used to reconstruct evolution of donor splice signals resulting in 298 inferred GT to GC conversion events compared to 40 GC to GT conversion events in primate and rodent genomes. Thus, there was substantial accumulation of GC donor splice signals during the evolution of mammals. Accumulation of GC sites might have been driven by selection for AS [195]. Several studies have dealt with the evolution of alternative splicing from the perspective of the evolution of splicing enhancers and silencers, and some signs of negative and positive selection have been detected [199-206].

Alternative splicing is one of the primary sources of 5'UTR transcript diversity, and in several reports the hypothesis has been put forward that these mechanisms might play an important role in orchestrating complex regulatory mechanisms within 5'UTRs [207-209]. Estimates of the number of genes with alternative 5'UTRs vary from 10% to 22% [179,210,211]. A genome-wide comparative study of 5'UTR sequences in primates and rodents revealed a much greater abundance of alternative splicing (and alternative transcription) than detected in the corresponding coding sequences, conceivably because 5'UTRs are not bound by constraints on protein structure that limit alternative splicing in coding regions [209]. Alternative regions of 5'UTRs contain numerous upstream AUG codons and short upstream open reading frames, consistent with the hypothesis that alternative events in 5'UTRs of mammalian genes contribute to the regulation of translation [209].

## Functions of introns

The non-adaptive population genetic perspective on the evolution of eukaryotic gene architecture implies that introns are devoid of function, at least originally. This conjecture is compatible with the numerous analyses indicating that, beyond the splice signals, intron sequences are subject to weak purifying selection at best, or evolve in a regime that is indistinguishable from neutral evolution [212,213]. However, this (nearly) neutral background of intron evolution does not rule out the possibilities that, first, the very presence of introns affects the regulation of expression of the respective genes (presumably through the interaction with the splicing machinery) and hence their function, and second, that many introns harbor specific functional elements. Indeed, there is abundant evidence that introns are often functional at both levels.

Potential functions of introns can be separated into three categories: i) functions associated with splicing, ii) generic functions of non-coding DNA, and iii) genes nested within introns. In addition, the possibility has been discussed that introns might act as ‘catalysts’ of evolution by facilitating intergenic recombination (this could be considered a variation on the theme of generic non-coding DNA functions). Experimentally demonstrated and potential functions of introns have been reviewed in detail [214,215]. Here we do not attempt a comprehensive coverage of this subject but rather briefly discuss several aspects that appear directly relevant for understanding evolution of introns and eukaryote gene structure.

### Functions of introns associated with splicing

Splicing occurs before mature mRNAs are transported from the nucleus to the cytosol by the nuclear export system. Numerous studies indicate that splicing and mRNA export are directly coupled (see reviews [32,35]). Evidence of such coupling initially came from the observation that mRNAs generated by splicing are more efficiently exported than their identical counterparts transcribed from a complementary DNA [216]. This effect of splicing on export was explained by the finding that spliced mRNAs (but not cDNA transcripts) are assembled into a distinct mRNP complex that promotes efficient export [32,35,216]. This complex, or at least some of its components, has been subsequently shown to assemble adjacent to newly formed exon–exon junctions [217]. The increased export efficiency of the spliced mRNP is thought to be due to recruitment of the mRNA export factor ALY to the mRNA during the splicing reaction [218,219]. The splicing factor UAP56, which interacts directly with ALY, plays a role in recruiting ALY to the spliced mRNA [220-222]. In a subsequent step, a hand-off occurs in which the ALY/TAP interaction is established, thus delivering the mRNP to the nuclear pore for export [221]. The numerous eukaryotes that possess only a few introns in the entire genome nevertheless retain a full-fledged or partially degraded spliceosome machinery [8,65,223], suggesting the possibility that the spliceosome might have functions other than splicing as such, perhaps primarily nucleocytoplasmic trafficking. However, the transport mechanisms for numerous intron-less transcripts are not well characterized, and the possibility remains that intron-less RNAs are recruited to the export machinery via a spliceosome-independent route [32,35]. Compatible with this hypothesis, UAP56 is required for export of both spliced and intronless mRNAs [220-222,224]. In metazoan intronless mRNAs, specific mRNA sequence elements are required for export, and some of these elements associate with members of the SR family of splicing factors which are thought to mediate export of the intronless mRNA [225,226]. The SR proteins could either recruit the conserved export machinery or play a direct role in export [226]. In both yeast and metazoans the export of intronless mRNAs also could be coupled to polyadenylation [32,35,226,227]. It has been shown that in mammalian neurons some retained introns are coupled with targeting of mRNA sequences to dendrites, apparently via so called ID sequences that represent a distinct class of SINE retrotransposons resident in the retained introns [228]. Thus, functionally relevant retention of intronic sequence might be a more general phenomenon than previously suspected.

The speed of splicing could be another important mechanism of gene expression regulation [27,28]. Analysis of minor, U12 introns (see above) suggested that their positions are conserved in orthologous genes from human and Arabidopsis to an even greater extent than the positions of the major, U2 introns [29]. The U12 introns, especially conserved ones, are concentrated in 5'-portions of plant and animal genes, whereas the U12 to U2 conversion occurs preferentially in the 3'-portions of genes. These results are compatible with the hypothesis that the high level of conservation of U12 intron positions and their persistence in genomes, despite the unidirectional U12 to U2 conversion, have to do with the role of the slowly excised U12 introns in down-regulation of gene expression [27-29,229].

As already pointed out above, introns in yeast ribosomal protein genes substantially affect the expression of these genes and contribute to the organismal fitness and stress response via mechanisms that are not yet well understood [57]. These seminal findings indicate that in many cases the regulatory functions of introns could be specific to a class of genes or even an individual gene. This conclusion is compatible with the results of an earlier study which has shown that yeast spliceosome can distinguish between different transcripts including related ones, such as paralogous ribosomal protein genes, thus providing a distinct regulation mode for expression of specific proteins [230].

### Introns as functionally important non-coding DNA sequences

Compared to prokaryotes, eukaryotes possess a much greater number of multidomain proteins that substantially contribute to the functional complexity of the eukaryotic cell [187,188,231-234]. Moreover, a striking feature of eukaryotic protein architectures is the wide spread of the so-called promiscuous domains that combine with other domains much more often than expected by chance [234,235]. The ‘exon theory’ posits that exon shuffling via recombination within introns is an important route of evolution that in particular is responsible for the diversity of the domain architectures of multidomain proteins [39,40,236]. In the specific case of vertebrate membrane receptor proteins, this hypothesis seems to be compatible with empirical observations: these proteins consist of multiple modules each of which typically is encoded by an individual exon [185,187,188]. However, in other classes of proteins, there is no strong preference for intron location between domains, so exon shuffling is unlikely to be a major, general mechanism of multidomain protein evolution [43,135,185,187,188,234].

Introns have the potential to serve as “enhancers” of meiotic crossing-over occurring within protein-coding genes because the probability of crossing over between segments of a coding sequence (exons) separated by long introns greatly increases compared to the same coding sequences in the absence of an intron [214,237]. This meiotic recombination between exons of two alleles of the same gene is likely to be a major factor of protein evolution through combining mutations from different alleles, “trying out” different combinations and avoiding accumulation of deleterious mutations within the same allele [1,214,237].

Trans-splicing is a special form of RNA processing whereby exons from two different primary RNA transcripts are joined end-to-end and ligated. The most common form of trans-splicing is spliced-leader (SL) trans-splicing where the leader is donated by a short SL RNA. The SL trans-splicing is widespread among some unicellular eukaryotes, in particular trypanosomes [238]. Other than trypanosomes, the only organisms known to heavily rely on SL trans-splicing for gene expression are nematodes. More than half of the pre-mRNAs in the *Caenorhabiditis* nematodes are trans-spliced to one of two short leader RNAs, SL1 or SL2. This process occurs at the 5' ends of pre-mRNAs, and it is essential for the efficient processing of polycistronic pre-mRNAs [35,239-242]. The patchy distribution of trans-splicing suggests that SL trans-splicing has evolved repeatedly among eukaryotic lineages and SL precursor RNAs have readily evolved from ubiquitous small nuclear RNAs that are involved in conventional splicing [243]. Several cases of trans-splicing between different pre-mRNAs (no SL RNAs are involved) have been identified in tunicates, mammals, flies and plants (reviewed by [214,242,244,245].

### Functional elements and genes within introns

Some introns contain various regulatory elements as well as sequences involved in chromatin structure formation such as scaffold-matrix attachment regions, although it remains uncertain whether intron sequences show any substantial enrichment for regulatory and structural elements compared to other non-coding DNA [214,246]. Some long introns, especially those in 5’-terminal regions of coding sequences, might be enriched for various regulatory elements, and consequently, could be subject to purifying selection [160,247-253]. Long introns in several genes of *Oikopleura* have been shown to contain key developmental regulators [131], and similar observations have been reported for genes involved in development of diverse metazoans [254-257] as well as associated “bystander” genes that are not known to be directly involved in development [258-261].

Many introns contain within their sequences various non-coding RNA genes, especially numerous genes for snoRNAs [262,263] and precursors of microRNAs [264,265]. Specifically, some short animal introns with hairpin formation potential, known as mirtrons, can be spliced and debranched into pre-miRNAs [266-268]. These pre-miRNAs are then cleaved by the RNase III enzyme Dicer and incorporated into typical miRNA silencing complexes [268,269].

A small fraction of introns contain nested protein-coding genes [270]. Comparative analysis of these nested genes in vertebrates, fruit flies and nematodes revealed substantially higher rates of gain of intron-embedded genes compared to loss [271]. However, the accumulation of nested gene structures is likely to represent an increase of organizational complexity of animal genomes via a neutral process given that there seem to be no functional links between the intron-contained genes and the ‘host’ genes [271]. Effectively, it seems that introns serve as neutral substrate that can be randomly colonized by various genes.

## Molecular mechanisms of intron loss and gain

Mechanisms of intron loss and gain remain poorly understood. A plausible, common mechanism for intron loss could be homologous recombination between cDNAs that are produced by reverse transcription and the genomic copies of the respective genes [65,67,107-110,112]. Intron gain/loss events must be associated with a transient phase of segregating alleles either carrying or lacking the intron within natural populations [49]. Until now, only 25 transient intraspecific intron presence-absence polymorphisms have been reported, one in *Drosophila teissieri*[272] and 24 in *Daphnia pulex*[70,96]. In *Daphnia*, recently gained intron sequences were frequently associated with short repeats, suggesting a role for double-strand break repair in intron gain [96]. Analysis of several closely-related fungi revealed 74 presence-absence polymorphisms of introns [273]. Examination of the positions of these introns has suggested that extensive intron transposition among unrelated genes is the major mechanism of intron gain in the analyzed fungal genomes [273]. The existence of large families of highly similar intron sequences in these genomes suggests that certain intron sequences are much more likely to be transposed than others and that specific sequence patterns might promote intron transposition [273].

Although transposition of introns could be an important factor of intron gain for some fungi, it appears to be a negligible route of intron evolution in nematodes, green plants, and Daphnia [66,94,96]. It is likely that intronization of (parts of) exons is an important source of new introns [110,114,274,275]. However, possible other routes of intron acquisition let alone their quantitative contributions in different groups of eukaryotes have not been characterized in any detail [72,275,276]. A striking case of massive intron gain has been discovered in the course of genome analysis of the marine picoeukaryotic alga *Micromonas pusilla*[277]. The introns of numerous *Micromonas* genes contain repeat sequences that are absent from orthologous genes in closely-related genomes. These abundant ‘introner’ elements (9904 introners total) were located within introns, extended nearly to donor and acceptor sites, and lacked known characteristics of transposable elements [277]. The high abundance of introner elements suggests that these elements are either functionally important or resistant to purging, or both. It should be noted that mechanisms of massive intron gain events (for example, in the earliest eukaryotes [148,275] could well be different from mechanisms of relatively slow intron acquisition process in various extant eukaryotes [66,94,96,273] which makes delineation of mechanisms of intron gains an even more difficult problem.

## Origin and evolution of spliceosomal introns: a synthetic concept

The evidence presented here and elsewhere [42,45,53,62,84] supports a ‘numerous introns early in eukaryote evolution’ view. The discovery of introns in jacobids [278] and other excavates [4,5] is compatible with this concept. Even more strikingly, approximately 60% of the introns in the parabasalid *Trichomonas vaginalis* occupy the exact position of an intron in an orthologous gene from at least one other eukaryotic lineage [279], and similar observations have been made for the free-living excavate *Naegleria gruberi*[6]. Most importantly, probabilistic reconstructions of intron gain and loss provide consistent and by now compelling evidence that ancestral eukaryotic forms including the LECA possessed intron-rich genes, with intron densities comparable to those in the most intron-rich modern organisms such as mammals [53,141]. These findings have fundamental consequences for our understanding of the evolution of eukaryotes and possibly of the ultimate origin of the eukaryotic cellular organization [46,280,281].

It appears likely that the emergence of the eukaryotic cell or the initial stages of its evolution involved, among other radical innovations, a catastrophic intron invasion (Figure 9) [46]. Structural similarities between the terminal regions of spliceosomal introns and those of self-splicing Group II introns (retro-transcribing elements) leave essentially no doubt in the existence of a direct evolutionary connection between the two classes of introns [282]. Moreover, the elements of Group II introns involved in the autocatalytic splicing reaction apparently also gave rise to the spliceosomal small RNAs [282,283]. Thus, at an early stage in the evolution of eukaryotes, an irreversible transition apparently took place from autocatalytic splicing to splicing mediated by a universal trans-acting catalyst (the spliceosome). This transition involved the split of the ancestral Group II intron structure into the catalytically inert spliceosomal introns and the catalytically active RNA moiety of the spliceosome that was also accompanied by the degradation of the reverse transcriptase open reading frame within introns [280].

It appears most likely that the Group II intron invasion was triggered by the establishment of the endosymbiosis between an α-proteobacterium and an archaeal host (Figure 9). Notably, α-proteobacteria typically contain in their genomes a relatively large number of Group II elements compared to other bacteria [284]. Upon the endosymbiont invasion of the archaeal host, the symbiont’s Group II introns might have been ‘unleashed’, in part due to repeated lysis of the symbiotic cells (the evolving mitochondria) [280]. At the fundamental evolutionary-theoretical level, the tolerance of the emerging eukaryotic cell to such an invasion could be potentially explained by a population bottleneck which severely limited the efficacy of purifying selection [50,280,285].

Indeed, it has to be emphasized that Group II introns are typical mobile elements that actively spread around the host genome when given a chance by weakness of purifying selection pressure.

However, at the mechanistic level, the adaptation of the early eukaryotes to the swarms of genomic parasites (if this is what introns are, Figure 9), which severely compromised the integrity of their genomes, an adaptation that apparently involved rapid evolution of the dauntingly complex spliceosome, remains an intriguing enigma. The intron invasion, probably spawned by the mitochondrial endosymbiont (Figure 9), could have led to a peculiar, intron-dominated genome architecture of the early eukaryotic, with up to 80% of the genomic DNA comprised of introns [286]. This genome structure could be sustainable only under a severe population bottleneck and might have critically contributed to the emergence of the principal features of the eukaryotic cell [46,286]. The evolution of the signature features of eukaryotic cell organization, such as the endomembrane apparatus including the nucleus, the nonsense-mediate decay system and the ubiquitin system, can all be conceptualized as multiple levels of defense against the deleterious effects of the intron invasion [46,172]. Furthermore, the early, mobile introns could have triggered the proliferation of multidomain proteins via homologous recombination between introns in different genes. Obviously, most of such events would be strongly deleterious but some might have created potentially useful domain combinations without losing much important information, and thus would be picked by selection. Introns also created the potential for controlled alternative splicing (see above), a mechanism that came to prominence at a later stage of eukaryotic evolution and made a crucial contribution to the evolution of complexity in multicellular organisms. To summarize, the intron invasion that was probably concomitant with the emergence of the first eukaryotic cells can and probably should be envisaged as one of the key factors of eukaryogenesis.

Evolution of exon-intron structure of eukaryotic genes had been long considered in the context of the “introns-early” vs. “introns-late” debate [39-42]. Although the original introns-early idea is hard to reconcile with the absence of spliceosomal introns (and the spliceosome itself) in prokaryotes and the absence of conserved intron positions in ancient eukaryotic paralogs (Figure 8) [148], this concept can be easily restated in more realistic (even if less dramatic) terms. Specifically, the entirety of the observations discussed above, strongly suggests that the spliceosomal introns originated from self-splicing Group II introns which invaded eukaryotic genes (or perhaps more precisely, genes of the archaeal host of the proto-mitochondrial endosymbiont) concomitantly with or at the latest shortly after the origin of the eukaryotic cell. As indicated by evolutionary reconstructions, subsequent evolution involved mostly lineage-specific loss of introns punctuated with a few episodes of new gains. Under this scenario, although there is no evidence of existence of modern-type spliceosomal introns (or spliceosomes) prior to the origin of eukaryotes, their ancestors were ancient mobile elements that probably co-existed with cellular life forms throughout their evolution or possibly even antedated modern cells [287]. Thus, although the ‘exon hypothesis’ and the original idea that the first genes contained multiple introns do not seem to be supported by any evidence, the evolutionary lineage leading to spliceosomal introns indeed could be as old as some of the first replicating genetic entities.

## Conclusions

The incentive to write this review was the conviction of the authors that, after 30 years of turmoil, a degree of clarity has been reached in the study of the evolution of eukaryotic gene architecture. This progress has been achieved through the combination of comparative analysis of numerous, diverse genomes of eukaryotes, probabilistic reconstructions of intron gains and losses, and the non-adaptive population genetic theory of evolution of genomic complexity. It now appears well established that evolution of eukaryotes as a whole as well as evolution of each of the eukaryotic supergroups started with intron-rich genomes with relatively weak, error-prone splice signals. The evolution of these ‘cumbersome’ ancestral genomes was predicated by population bottlenecks that accompany evolutionary transitions and entail weak purifying selection that is incapable of purging introns or evolving efficient splicing. Subsequent evolution of eukaryotes followed the divergent paths of genome streamlining which led to elimination of the majority of introns and tightening of the splice signals, or genome complexification which involved evolution of functional alternative splicing and other intronic functions. The streamlining route is characteristic of many lines of descent that enjoyed evolutionary success and reached large effective population size (primarily unicellular eukaryotes and some fungi), whereas the forms that never achieved high efficiency of purifying selection (primarily multicellular animals and plants) followed the path to complexity.

The elucidation of the general scenario of evolution of eukaryote gene architecture by no account implies that the main problems in the study of intron evolution and function have been solved. Quite the contrary, fundamental questions remains wide open.

What are the mechanisms of intron loss and gain? There is very little direct evidence of any. A consensus seems to exist regarding the role of reverse transcription in intron loss although even this mechanism badly needs experimental corroboration. As for the mechanisms of intron gain, indications of the involvement of double-strand break repair notwithstanding, the study of this key problem has not even started in earnest.

What are the sources of new introns? It is clear that duplication of pre-existing introns is a negligible route of intron evolution in many eukaryotic lineages although it seems to be important in some, whereas intronization of (parts of) exons appears to be a significant contribution throughout the evolution of eukaryotes. However, possible other routes of intron acquisition let alone their relative quantitative contributions remain unknown.

What is the general role of introns in gene expression and function (if any) and why is it the case that new genes (such as those acquired from chloroplasts in plants) are saturated by introns at an apparent high rate? And a related question: why do a handful of introns (and with it the spliceosome, sometimes partially degraded) survive in the great majority of even the most streamlined eukaryotes? At best, only most general and largely speculative answers to these key questions are currently available. These are hard questions, and the only hope to obtain satisfactory answers is to combine comprehensive phylogenomic analysis with population genetic models and extensive experimentation.

## Competing interests

The authors declare that they have no competing interests.

## Authors’ contributions

IBR, LC, MC and EVK wrote the manuscript which was read, edited, and approved by all authors.

## Reviewers' comments

**Reviewer #1: Dr. I. King Jordan**, Georgia Institute of Technology

Igor Rogozin and colleagues have written a comprehensive, synthetic and compelling review mainly covering the ‘evolution’ of spliceosomal introns. As in Darwin’s famous tome, the notion of ‘origin’ is actually given short shrift, but I will come to that point later. In any case, this ambitious work benefits from the broad perspective that the authors have gained over years of investigating the subject. Along with this perspective comes some inevitable bias, or perhaps it is more fair to say a favored world-view, as described below with respect to the authors position on ‘introns-kind-of-early’. But this does not represent a liability of the work in my opinion; the authors are clearly entitled to their views, and the conclusions they draw appear to be both nuanced and well-supported by the data. They cover a lot of ground herein and strike a nice balance between thoroughly reviewing the relevant literature and elucidating the most salient points from the large body of work on the subject. One of the major conclusions of this review relates to a resolution, or compromise really, of the ‘introns-early’ versus ‘introns-late’ debate that consumed the field for many years. The authors champion a merger of these two hypotheses into the ‘many introns early in eukaryotic evolution’ view, whereby the earliest eukaryotic lineages contained genomes that were already loaded with many introns and subsequent evolution was dominated by intron loss.

The parts of the review that cover the origin of spliceosomal introns are the most speculative and least supported. This is not a critique per se; it may simply be the case that the study of origins must always be more speculative than the study of evolution. According to the ‘many introns early in eukaryotic evolution’ hypothesis, the earliest eukaryotic genomes were formed via massive intron invasion that resulted in genomes consisting of up to 80% intronic DNA. Crucially, the authors hold that this invasion was probably facilitated by low effective population sizes and the corollary weak purifying selection, following the influential Michael Lynch model for the non-adaptive evolution of eukaryotic genome complexity. This model accounts for population level dynamics but neglects the internal dynamics of the genome. If spliceosomal introns indeed evolved from Group II introns, as the authors maintain, then the initial intron invasion of eukaryotic genomes would have been driven, to some extent, by a kind of selfish genetic element with its own internal drive mechanism to replicate within the genome. In theory, such selfish replicators can efficiently increase in copy number even in the face of a selective cost to the host. Therefore, the early origin of introns may be attributed to an active internally driven process, rather than a solely passive drift related process, i.e. a mechanism akin to the molecular-drive concept of Gabriel Dover or the mutation bias emphasized by Arlin Stoltzfus. Such an active replicative process inside the genome could have actually outpaced selection’s ability to contain it. The authors actually touch on this notion, when they speculate as to whether introns are genomic parasites and how the host may have evolved the spliceosome as an adaptive response to intron invasion, but an explicit connection between their selfish drive to replicate and the origin of introns is not made.

Authors’ response: *We agree on all accounts. Yes, it comes with the territory: discussion of origins is inevitably more speculative than the analysis of subsequent evolution. More importantly, the role of the active mobility of Group II introns certainly must not be under-appreciated, and we explicitly point this out in the revised manuscript: ‘Indeed, it has to be emphasized that Group II introns are typical mobile elements that actively spread around the host genome when given a chance by weakness of purifying selection pressure.’*

One specific suggestion as to how the work can be improved relates to the abstract. Currently, the abstract is very short and concise, whereas the manuscript is rather long and presents a lot of material. I think it would be helpful to provide a more detailed abstract that specifically enumerates the authors’ most important points, something more like of a summary of the last two sections of the manuscript.

Authors’ response: We fully agree, the original short abstract resulted from a misunderstanding regarding the limits on abstract length in review articles. In the revised article, the abstract was substantially expanded.

**Reviewer #1: Dr. I. King Jordan**, Georgia Institute of Technology (additional comment on the revised version of the manuscript)

I have re-reviewed the manuscript of Rogozin et al. I am satisfied with the changes made, for the most part, and I recommend that the paper be accepted for publication in Biology Direct after the following point is addressed.

I would like the authors to elaborate just a bit on their response the first comment that I made, in particular with respect to the connection between Group II intron dynamics and the evolution (emergence) of introns. I think I may have rambled a bit in my original comment and was not explicit enough. I would urge the authors to have a look at the manuscript of Donal Hickey from Genetics (Hickey 1982 101; 519), which makes the point much better than I did in my comment. The population genetics models in the manuscript may be a bit simplistic by this time, but I think the ideas contained therein are highly relevant to their own work. In particular, Hickey makes an explicit connection between the genome dynamics of mobile elements, host selection pressure and the evolution of introns. The basic idea is that mobile genetic elements can spread in a population even in the face of a fitness cost to the host, and this kind of process could have resulted in the emergence and spread of introns. Below, I provide a comment in response to the authors' response to my first comment in an attempt to facilitate further discussion and consideration of this issue.

Authors' response: We agree on all accounts. Yes, it comes with the territory: discussion of origins is inevitably more speculative that analysis of subsequent evolution. More importantly, the role of the active mobility of Group II introns certainly must not be under-appreciated, and we explicitly note in the revised manuscript: 'Indeed, it has to be emphasized that Group II introns are typical mobile elements that actively spread around the host genome when given a chance by weakness of purifying selection pressure.'

Response: I would like the authors to further consider the possibility that mobile elements (such as Group II introns) can increase in frequency in a population, even when they impose a fitness cost on their host organisms, owing to the fact that a replicative transposition process results in a biased transmission rate relative to host genes. This idea was introduced by Donal Hickey 30 years ago, and he also connected this point to the evolution of introns (Hickey 1982 Genetics 101: 519). In other words, it is not simply a matter of weak purifying selection allowing active spread of the elements, but an effect of the element mutational dynamics introducing directional bias in the evolutionary process. This idea is very much analogous to the notion that mutation bias in the broader sense can be a cause of direction in evolution (e.g. see Yampolsky and Stoltzfus 2001 Evol Dev 3: 73).

Authors' response: *We agree that mutational dynamics of selifhs element could be an important driver of their spread. We think that once this additional exchange with the reviewer is published, the emphasis on this issue will be adequate.*

**Reviewer #2. Dr. Tobias Mourier**, University of Copenhagen (nominated by Dr Anthony Poole)

This review provides a comprehensive overview of the current knowledge of intron evolution in eukaryotic genomes.

The advent of numerous eukaryotic genomic sequences has consistently supported the 'many introns early in eukaryotic evolution' concept, as evident from the manuscript. But surely this hypothesis is not a merger from the introns early/late/first ideas (as the authors write in the "Intron-early, introns-late, introns-first …" section). All recording of spliceosomal intron features comes from eukaryotic genomes, and regardless of how many eukaryotic genomes are sequenced, extant spliceosomal intron features will never allow one to synthesize past LECA.

In the end of the manuscript, the authors present a scenario proclaiming that an intron-rich LECA is not inconsistent with the introns-late hypothesis. This is not a problem, but the structure of the manuscript may give the impression that this is a conclusion (or synthesis) directly from the current knowledge of eukaryotic gene architecture (that is nicely reviewed in the preceding text).

Authors’ response: Actually, we do believe that the synthesis we present in the section of the review preceding the Conclusions follows from the comparative genomic results reviewed in the preceding sections. Certainly, not all parts of the article directly contribute to this synthesis: for instance, the discussion of the functional roles of introns is only tangentially relevant here albeit important in other respects. Nevertheless, we do maintain that in this section we present major implications of the comparative genomic study of eukaryotic gene structure.

The review presents an overview of the comparative approaches taken to delineate intron-exon structures during evolution. The basis for such comparative analyses is well-aligned sequences around splice sites. If intron-exon structures to some extent evolve via mechanisms such as alternative splicing and intronization of exonic sequence, should this not result in sequences that are unlikely to meet the criteria for being included in the above analyses? I think it would be relevant to discuss the implications of this.

Authors’ response: This issue is discussed in the section ’Evolutionary conservation of intron positions and routes of gene architecture evolution of eukaryotes’.

Section "Functional elements and genes within introns"

When discussing intronic RNA genes, I'm surprised there is no mentioning of the classical connection between vertebrate snoRNAs and introns (and perhaps even the existence of genes with non-coding exons and introns encoding snoRNAs, (e.g. Tycowski et al., Nature 1996).

Authors’ response: Yes, this certainly is an important theme, and we added it to the section ‘Functional elements and genes within introns’.

Very minor points:

Page 11: "whereas the remaining 6 Xist" rather than "whereas remaining 6 Xist"

Page 14: "and so does the strength" rather than "and so does and the strength"

Page 18: should "introns are inserts or fixed" read "introns are inserted or fixed"?

Authors’ response: All corrected, we appreciate the reviewer’s attention to these points.

**Reviewer #3. Dr. Manuel Irimia**, University of Toronto (nominated by Dr Anthony Poole)

Rogozin et al. have put together an impressively comprehensive review on the origin and evolution of splicesomal introns that will certainly become a major reference in the field. Overall, I found it easy and entertaining to read, as well as informative. I have only a few comments and suggestions, often regarding further literature, that I hope can help to improve the piece (listed according to their appearance in the main text):

Authors’ response: We appreciate Dr. Irimia’s close attention to the details of this article. As detailed below, we found most of the suggestions fully pertinent and modified the manuscript accordingly.

1) P3: The paragraph on splice site consensus sequences could provide a more detailed portrait of canonical intron signals across eukaryotes. For example, not all eukaryotes have polyT tracts between the branch point (BP) and the 3’ AG, and some fungal species even have polyT tracts upstream the BP (see Bon et al., Nucleic Acids Res 2003; Irimia and Roy, PLoS Genetics 2008). Also, some extremely intron-poor species intriguingly have strict GTATGT as consensus 5’sequence (including yeast), which may be worth pointing out. Finally, the 3’ consensus is closer to YAG than to CAG, at least in most species.

Authors’ response: We added discussion of this issue to the revised text.

2) P5: I found the (exciting) discussion on the ancestrality of U2 vs. U12 too short and a bit imbalanced. Personally, I think it is a good idea that the authors give their authoritative opinion/preference on this kind of discussions, but the opposite arguments should also be presented extensively. In this case, I think the arguments supporting an ancestral origin of U12 (i.e. lack of evidence for conversion from U2 to U12, argued higher similarity of U12 to type II introns, etc.) should be fully developed.

Authors’ response: In our view, the questionable greater similarity of U12 introns to Group II introns does not immediately imply ancestral status of U12 introns. We added to the text ‘it might be tempting to speculate that the ancestral introns were of the U12 type (for example, see discussion by the reviewer #3 below) but have been subsequently converted to U2 introns.’

3) P8: Pleiss et al. (PLoS Biol 2007) may be added supporting a global regulatory function of introns in yeast.

Authors’ response: We added discussion of this important work to the section ‘Functions of introns associated with splicing’.

4) P9: I missed a more comprehensive and complete review of the literature on the genome-wide dynamics of intron gain and loss in this section. For example, on the general slow paucity of intron gain, I missed references on vertebrates (Loh et al., MBE 2008; and actually ref 72 is incorrect: Coulombe-Huntington and Majewski, Genome Res 2007), flies (actual ref 72), plants (Roy and Penny, MBE 2007), apicomplexa (Roy and Hartl, Genome Res 2006; Roy and Penny, Genome Res 2006), Entamoeba (Roy et al., MBE 2006), Fungi (Nielsen et al., Plos Biol 2004; Stajich et al., Genome Biol 2007; in Aspergillus (Zhang et al., JME 2010)). On the opposite side: tunicates (Seo et al., Science 2001; Edvardsen et al., JME 2004), diatoms (Roy and Penny, MBE 2007), mitochondrial transfers (Ahmadinejad et al., BMC Evol Biol 2010). Given the overall level of comprehensiveness and detail of this review and that, as I said above, it is very likely to become a major reference in the field, I think it would be important to cite all relevant references in the main text, in particular from such an important and prolific subtopic.

Authors’ response: There is indeed a lot of evidence on specific events in individual lineage. We appreciate their importance but it is hardly possible to discuss ‘everything’ in detail. That said, the revised version of the review cites all the references pointed out by the reviewer.

5) P9: when commenting on ref 61, the use of the word “dispute” may give the impression that there is an ongoing controversy or a difference in opinions between the authors, which I guess is really not the case. Ref. 74 showed that most reported gains in ref. 61 were indeed losses by adding more species to the analysis that were not available by the time of the original study. This may not be clear to general readers that have not followed the specialized literature.

Authors’ response: We added this explanation to the text.

6) P11: the authors may wish to mention here the recent work by Cabili et al. (Genes Dev 2011), which describe >8,000 lincRNA genes, with an average of ~1.9 introns per Kbp and that are extensively alternatively spliced, with 2.3 isoforms per gene.

Authors’ response: We added a brief description to the text.

7) P14: I was quite surprised to read that the sequences at the 3’ of the intron behave completely different from those at the 5’. Many of the extremely intron-poor species (although not all, in this case) that show strict 5’ splice site consensus also have very strict BPs, and sometimes even very constrained branch-point-to-AG distances (Irimia and Roy, Plos Genetics 2008). I guess this apparent contradiction is due to the fact that these species are all missing from the analysis by Iwata and Gotoh (represented in Figure 5), which is strongly biased towards multicellular organisms, and I suspect that the inclusion of the intron-poor species would fully disrupt the observed negative correlation. In my opinion, this section should be modified to give a more complete view of the evolution of the 3’ intronic signals (more like 3-4 qualitatively different behaviors related to, but not fully determined by, intron densities). Also, I recommend removing Figure 5 or making a new one using a more complete eukaryotic taxon sampling.

*Authors’ response: We added a list of species to the legend. Robust estimation of the information content require hundreds of splice signals, so it is impossible for the extremely intron-poor species. This is why these species are missing from the analysis of Iwata and Gotoh, and accordingly, from our Figure*5*. We believe that it is fully legitimate to present only the data for those organisms that possess enough introns for meaningful statistical analysis. Furthermore, there is no contradiction at all between the observation that some extremely intron-poor species possess strict 5’ splice site and also have very strict BPs and the positive correlation between the strength of the donor splice signal and the combined strength of the branch point signal + the acceptor splice signal emphasized in the present article.*

8) P14: also related to splicing signals, it would be interesting to include a comment on the effect of intron size on splicing signals (long introns have stronger boundaries, species with extremely short introns often have very weak signals (e.g. paramecium, *B. natans* nucleomorph), etc.).

Authors’ response: The effect of intron size is complicated. We added discussion of this issue to the section on ‘Evolution of splicing signals, protosplice sites, and intron phase distribution.’

9) Figure 6 may be a bit unclear and “too raw” for non-specialists.

Authors’ response: We included an additional explanation in the legend: “An excess of protosplice sites in phase 0 is noticeable, however the ‘protosplice site’ hypothesis, which posits that introns are randomly inserted into protosplice sites, is unable to fully explain the observed over-representation of phase 0 introns.”

10) P26: the authors may want to point out from the beginning that the “two competing hypothesis” they present are not necessarily mutually exclusive.

Authors’ response: Added to the text as suggested.

11) P28: ref. 158 also concludes that alternative splicing has emerged early in eukaryotic evolution, so it should be cited along with ref. 166 and not with 28.

Authors’ response: Modified as suggested.

12) P31: more references may be added supporting the low conservation of alternative splicing in mammals (currently only one, from 2003, is given, but several studies have reached similar conclusions). Similarly, many other studies have dealt with the evolution of alternative splicing from the perspective of the splicing signals, not only regarding GC splicing donor sequences (e.g. evolution of ESEs and ESSs (Parmley et al., MBE 2006; Ke et al., Genome Res 2008; Irimia et al., PLoS One 2009) and their polymorphism in human populations (Stallings-Mann et al., PNAS 1996; Stanton et al., PNAS 2003; Fairbrother et al., PLoS Biol 2006; Carlini and Genut, JME 2006; Coulombe-Huntington et al., Plos Genetics 2009).

Authors’ response: A brief discussion and references added as suggested.

13) P32: perhaps the section “Functions of introns” would fit better before the section on alternative splicing (since the latter is one of those functions).

Authors’ response: Alternative splicing is not exactly a function of introns, rather a mechanism of modulation of protein and RNA function. In the functional section we addressed specific functions of intron sequences. This might be debatable but we consider the original order of the sections acceptable.

14) P33: the authors may want to add that some spectacular, functional exceptions are known to the general case that splicing occurs before mRNA is exported to the cytoplasm. For example, Buckley et al. (Neuron 2011) describe the case of some transcripts with retained introns, which drive subcellular location of the transcripts to the dendrites due to the presence of a particular transposable element within their sequence.

Authors’ response: We appreciate the reviewer bringing our attention to this exiting work. Cited and briefly discussed.

15) P34: the catalog of U12 introns by Alioto (Nucleic Acids Res 2007) could be referenced here.

Authors’ response: Cited as suggested.

16) P35: I think it could be useful to make a clearer distinction between Splice Leader (SL) trans-splicing and trans-splicing between two different genes from the beginning of the paragraph (I found it a bit confusing now). Also, the authors may wish to cite a very elegant analysis searching for trans-splicing in Drosophila using RNAseq on hybrids (McManus et al., PNAS 2010).

Authors’ response: We agree and have included a brief discussion and references as suggested.

17) P36: in this subsection I missed a paragraph on the (predictable and predictive) association between long introns and the presence of functional elements. For example, Denoeud et al. (Science 2011) found that the few genes with long introns in Oikopleura are enriched for key developmental regulators, and that those introns likely contain regulatory information. This has also been observed for many other developmental genes across metazoans [e.g. Shh (Muller et al., Development 1999), FoxP1 and Dach (Sandelin et al., BMC Genomics 2004); Gli3 (Abbasi et al., PLoS One 2007), Meis genes (Irimia et al., GBE 2011), etc.] and for associated non-developmental genes (“bystander” genes) (e.g. Woolfe et al., PLoS Biol 2005; McEwen et al., Genome Res 2006; Kikuta et al., Genome Res 2007; Engstrom et al., Genome Res 2007), with exciting implications for the evolution of genome architecture. Also, supporting the presence of regulatory elements, higher sequence conservation is often found in longer introns (Bergman and Kreitman, Genome Res 2001; Parsch, Genetics 2003; Haddrill et al., Genome Biol 2005; Marais et al., Genetics 2005; Halligan and Keightley, Genome Res 2006; Parsch et al., MBE 2010).

Authors’ response: Brief discussion and references included as suggested.

18) P38: the authors may add the report by Curtis and Archibald (Curr Biol 2010) to the list of different sources of spliceosomal introns.

Authors’ response: Cited as suggested.

**Reviewer #4. Dr. Fyodor Kondrashov**, Center for Genome Regulation, Barcelona

This is a straightforward and extensive review of everything that is known about the evolution of introns and then some more. I do not have much to add in addition to what the authors have already said. The only thing that I am left wondering about after reading this review is whether or not the authors think that Group II introns in LECA were involved in the transport of mitochondrial precursor genes into what is now the cytoplasm across the novel intracellular membrane. In light of the previous reviews I would leave it up to the authors to space and moderate the level and format of speculation, even though I believe that the nice synthesis the authors have produced make the review more interesting and useful.

Authors’ response: We appreciate this comment. We are not entirely clear about the exact meaning of the reviewer’s idea regarding mitochondrial genes. Is this about transfer of genes from the mitochondrial to the nuclear genome? If so, the possibility of involvement of the reverse transcriptase activity of Group II introns is intriguing but in the absence of specific evidence, one would think the main route was DNA recombination.
